# Characterization of mouse serum exosomal small RNA content: The origins and their roles in modulating inflammatory response

**DOI:** 10.18632/oncotarget.17448

**Published:** 2017-04-27

**Authors:** Xin Zhou, Zinan Jiao, Juling Ji, Shuyuan Li, Xiaodi Huang, Xiaoshuang Lu, Heng Zhao, Jingwen Peng, Xinya Chen, Qiuhong Ji, Yuhua Ji

**Affiliations:** ^1^ Institute of Immunology, College of Life Science and Technology, Jinan University, Guangdong, China; ^2^ Department of Pathology, Medical School of Nantong University, Nantong, China; ^3^ Stanford University School of Medicine, Stanford, California, USA; ^4^ Key Laboratory of Neuroregeneration of Jiangsu and Ministry of Education, Nantong University, Nantong, China; ^5^ Department of Neurology, Affiliated Hospital of Nantong University, Nantong, China

**Keywords:** mouse serum exosomes, small RNA sequence, miRNA, origins, inflammation

## Abstract

In the last decade, although studies on exosomal microRNAs (miRNAs) derived from serum and other body fluids have increased dramatically; the contents and biological significance of serum exosomes under normal conditions remain unclear. In the present study, we profiled the small RNA content of mouse serum exosomes (mSEs) using small RNAseq and found that fragments of transfer RNAs (tRNAs) and miRNAs were the two predominant exosomal RNA species, accounting for approximately 60% and 10% of mapped reads, respectively. Moreover, 466 known and 5 novel miRNAs were identified from two independent experiments, among which the five most abundant miRNAs (miR-486a-5p, miR-22-3p, miR-16-5p, miR-10b-5p and miR-27b-3p) accounted for approximately 60% of all the aligned miRNA sequences. As inferred from the identities of the well known cell- or tissue-specific miRNAs, mSEs were primarily released by RBCs, liver and intestinal cells. Bioinformatics analysis revealed over half of the top 20 miRNAs by abundance were involved in inflammatory responses and further in vitro experiments demonstrated that mSEs potently primed macrophages towards the M2 phenotype. To the best of our knowledge, this is the first study to profile small RNAs from mSEs. In addition to providing a reference for future biomarker studies and extrapolating their origins, our data also suggest the roles of mSEs in maintaining internal homeostasis under normal conditions.

## INTRODUCTION

Exosomes are 30–150 nm membrane-bound vesicles that are formed in endosomal multivesicular compartments and secreted into the external milieu when these compartments fuse with the plasma membrane [1, 2]. Almost any cells can synthesize and release exosomes into the body fluid circulation [3]. These circulating exosomes carrying the specific contents from their host are promising sources for identifying novel none-invasive diagnostic and prognostic biomarkers, especially for those tissues or organs that are not easily accessible by traditional tissue biopsies, such as visceral neoplasm and the brain [4–6].

In the last decade, numerous studies have been attempt to identify biomarkers from circulating nucleic acids, especially miRNAs [7]. Exosomes are regarded as the predominant form of circulating miRNAs [8], but understanding of their miRNAs and other small RNAs is still very limited. This deficiency has already seriously hampered research concerning circulating tumor biomarkers, as many of these reported miRNAs are highly expressed by one or more blood cell types rather than tumor–specificity [9]. To improve the accuracy and efficiency of biomarker discovery, clarification of the miRNA profile of serum exosomes is urgently needed. This information will help to elucidate the potential origins of circulating exosomes and reveal the extent to which certain cells or tissues contribute to the pool of circulating exosomes under normal conditions.

Other than their miRNA content, an even more active area of exosomes research is their roles in intercellular communication. Since the finding that exosomes carry genetic materials, mainly mRNAs and miRNAs, and mediate genetic exchange between cells [10], accumulating studies have described the multiple roles and functions of exosomes derived from different cells and tissues. For instance, chronic lymphocytic leukemia-derived exosomes actively promote disease progression [11], and mesenchymal stem cell-derived exosomes improve heart function after ischemic injury by promoting angiogenesis and anti-inflammation [12]. The blood is a physiological fluid for exosomes circulation in the body and contains billions of exosomes per microliter [13]; however, the biological functions of serum exosomes under normal or pathophysiological conditions remains unclear, possibly due to their uncertain origin and complex contents. MicroRNAs are considered as the main functional components of exosomes, and therefore, profile the miRNA and other RNA compositions of serum exosomes will contribute to our understanding of their biological significance.

Next-generation sequencing (NGS) is the best way to comprehensively and quantitatively analysis exosomal RNA content and has been successfully applied to profile exosomal RNA from multiple cultured cells [14] and human plasma [15]. Mice are the most frequently used animals for basic and clinical studies. In the present study, we isolated and characterized exosomes from mouse serum and profiled their small RNA contents by NGS. Furthermore, based on the obtained miRNA profile and existing knowledge regarding tissue and/or cell specific miRNAs, we attempted to determine the potential sources of these circulating exosomes. Finally, the potential biological functions of mSEs were predicted by bioinformatics analysis and evaluated using *in vitro* experiments. These data will not only contribute to biomarker studies on serum exosomal miRNAs, but also will deepen our understanding of their biological significance.

## RESULTS

### Isolation and characterization of mSEs

Considering the limited volume of mouse serum and low yield of repeated ultracentrifugation, ExoQuick, a method based on precipitation, was selected to isolate mSEs. Consistent with the fundamental characteristics of exosomes [16], TEM analysis showed the obtained particles were spherical structures with diameter approximately 100 nm (Figure 2A). NTA measurement further confirmed that the mode size of these particles was 107 nm, with less than 5% of the particles were larger than 150 nm (Figure 2B and 2C). NTA analysis also showed that the particle concentration at a 1,000-fold dilution was approximately 7 × 10^8^ particles/ml.

**Figure 1:**
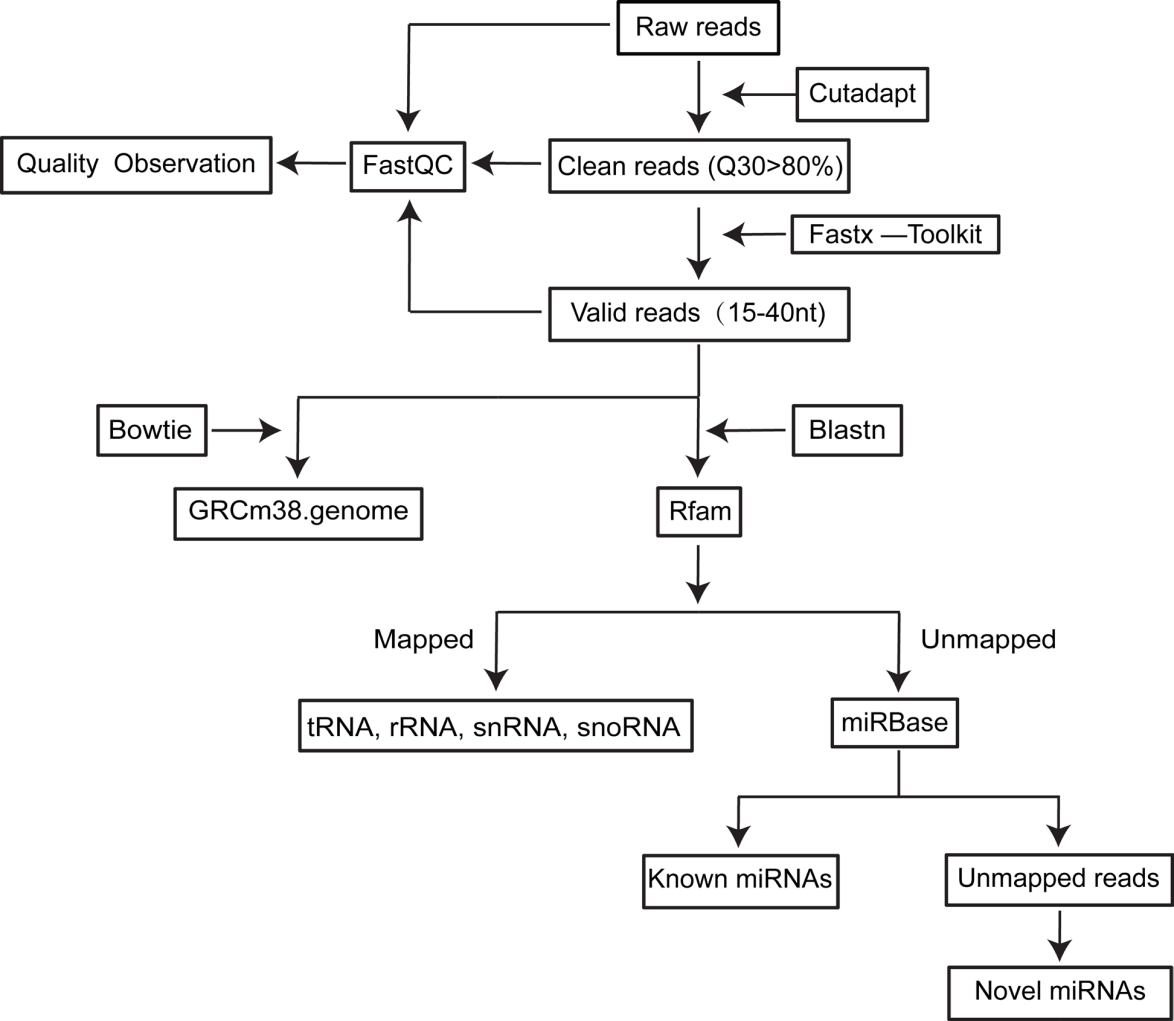
Flow chart for small RNAseq data processing

**Figure 2:**
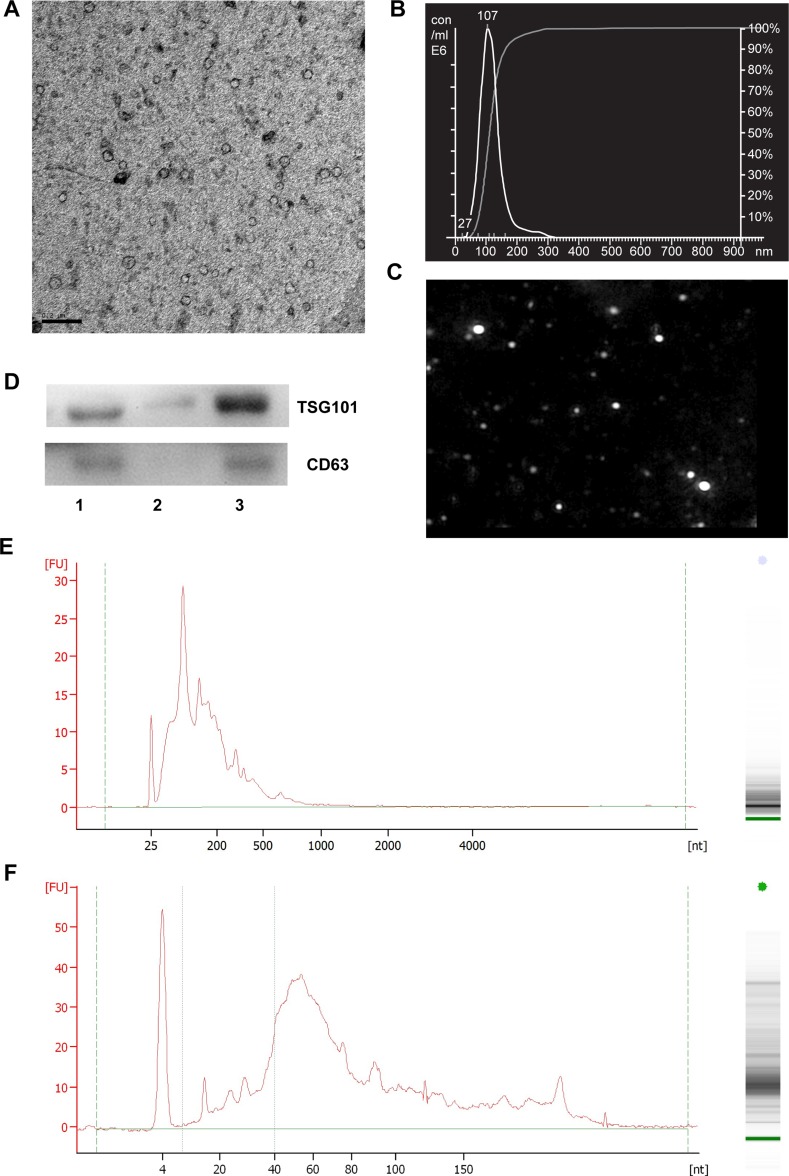
Characterization of exosomes isolated from mouse serum by Exoquick precipitation (**A**) The TEM image depicts the spherical morphology of the isolated particles, bar = 200 nm. (**B**) The NTA analysis plot illustrates the size distribution and concentration of the isolated particles. The indicated concentration is the value for samples that were diluted 1,000-fold. (**C**) NTA analysis image corresponding to the data in (B). (**D**) The expression of TSG101 and CD63 in the isolated particles as determined by Western blotting. Lanes 1 to 3 are serum, exosomes-depleted serum and serum exosomes, respectively. The size distribution of mSEs RNAs as detected by Bioanalyzer and RNA chip (**E**) and small RNA chip (**F**). The images are the representatives of three or more independent experiments.

Western blot was performed to determine the expression of two exosomal markers TSG101 and CD63. As shown in Figure 2D, both mouse serum and isolated exosomes expressed these two markers, but bands for these two proteins were absent or barely detectable in the exosomes-depleted serum. These results not only confirmed the expression of exosomal markers from isolated particles, but also suggested the high efficiency of ExoQuick in recovering mSEs.

The size distribution and concentration of RNA extracted from the isolated particles were determined using the Bioanalyzer. Total RNA chip analysis showed that the lengths of the major mSEs RNAs were less than 200 nt, with a small portion of RNAs greater than 500 nt in length (Figure 2E). Small RNA chip analysis revealed a peak at approximately 60 nt, and approximately 30% of the RNA had lengths indicative of miRNA species (4–40 nt) (Figure 2F). The exosomal RNA yields were approximately 30 to 40 ng per 250 μl serum. In short, these data demonstrated that particles obtained from C57BL/6 mouse serum by ExoQuick precipitation conformed to the major characteristics of exosomes [16], and therefore this isolation method was used in the subsequent experiments.

### Profiling the small RNA contents of mSEs

Small RNA sequencing was performed to profile the small RNA content of the mSEs. To evaluate the biological replication, two small RNA sequencing libraries were prepared individually from two batches of total exosomal RNA (7–9 mice per batch). The data produced during library construction are shown in Supplementary Figure 1.

From 2 libraries, 13,239,168 and 12,332,524 raw single-end reads were generated, respectively (Table 1). After the pre-processed and trimmed procedures were completed as described in the methods and illustrated in Figure 1, 11,375,272 and 9,789,718 valid reads with insert lengths between 15 to 40 nt were obtained, respectively. More than 80% of these valid reads could be aligned to the mouse genome (GRcm38), and less than 2% were unique reads, reflecting the high redundancy of the small RNA species in mSEs.

**Table 1:** Sequence data summary statistics

Description	Sample A	Sample B
Raw reads	13,239,168	12,332,524
Clean reads	12,385,336	12,323,254
Valid reads (Percentage of clean reads)	11,375,272 (92%)	9,789,718 (79%)
Unique valid reads (Percentage of valid reads)	191,410 (1.68%)	192,560 (1.97%)
Aligned reads (Percentage of valid reads)	9,498,447 (83.50%)	8,462,450 (86.44%)
Unique Aligned reads (Percentage of Unique reads)	118,158 (61.73%)	106,128 (55.11%)
miRNA reads	1,248,056	1,148,525
(Percentage of Aligned reads)	11.84%	11.73%
Unique miRNA reads	2418	2060
(Percentage of Unique reads)	1.26%	1.06%

**Figure 3:**
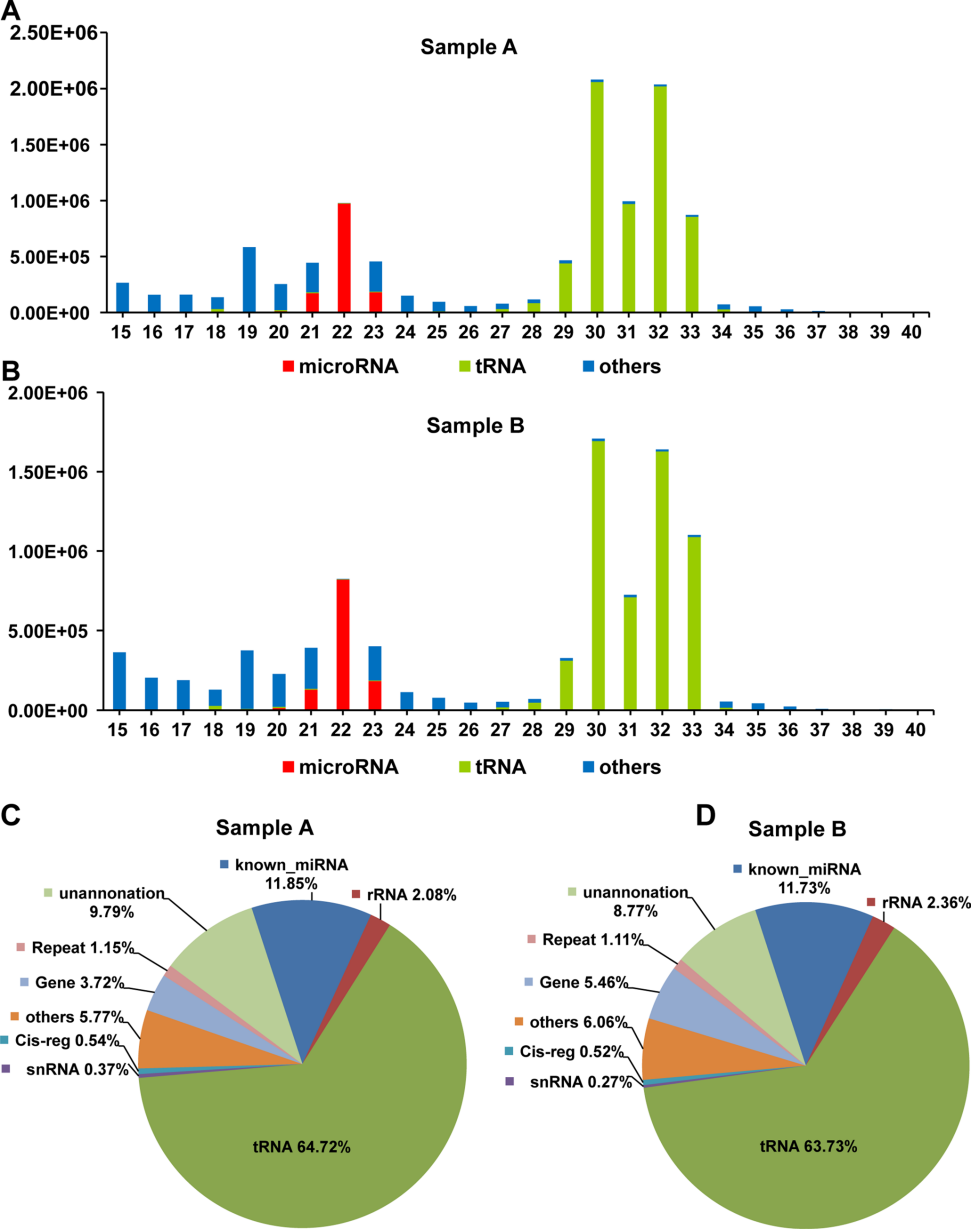
The length distribution and frequency of annotated reads Length distribution of the annotated reads, and the identified miRNAs and tRNAs from samples A (**A**) and B (**B**). Pie chart of annotated small RNA species and their percentages in samples A (**C**) and B (**D**).

The most significant feature of the size distribution of these annotated small RNAs of mSEs was the two peaks at 22 nt and 30 nt (Figure 3A and 3B). Transfer RNAs and miRNAs were the two major small RNA species present in the mSEs. The tRNAs accounted for over 60% of the annotated reads, and those for miRNAs accounted for approximately 10% (Figure 3C and 3D). By overlaying the length distributions of the identified miRNAs and tRNAs onto those of the annotated reads, we found that the miRNAs were predominantly distributed between 18–27 nt, whereas the tRNAs were mainly distributed between 29–33 nt, which corresponded exactly to the abovementioned two peaks (Figure 3A and 3B). Very low proportions of ribosomal RNA (rRNA), small nuclear RNA (snRNA), small nucleolar (snoRNA) and piwi-interacting RNA (piRNA) were also detected in the mSEs (Figure 3C and 3D). No significant statistical difference was observed between the biological duplicates in their percentages of these different small RNA species.

### Known miRNAs in the mSEs

**Figure 4:**
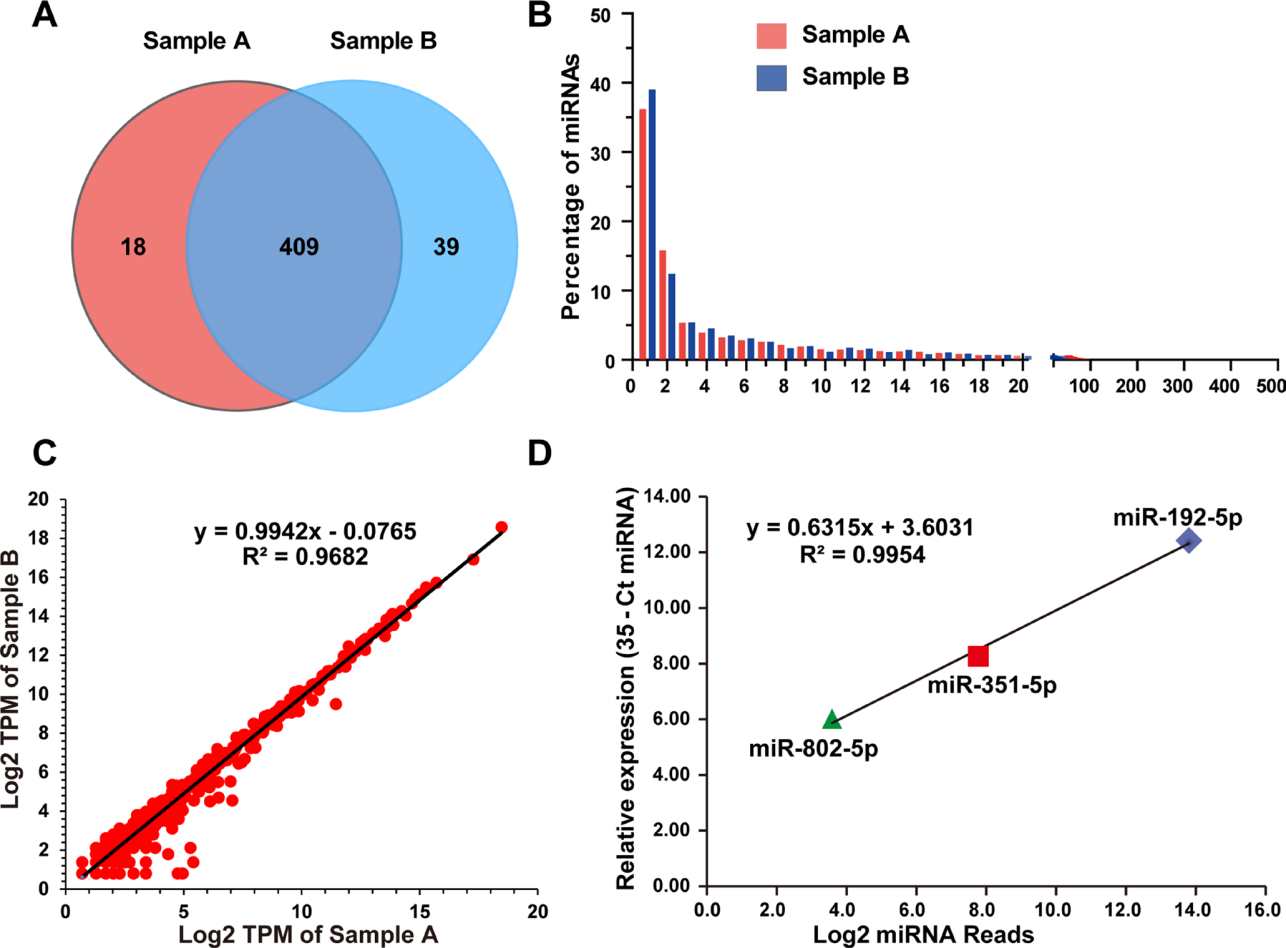
Profiling of mouse serum exosomal miRNAs (**A**) The venn diagram depicts the common and unique miRNAs identified in Samples A and B. (**B**) The histogram illustrates the percentage of each identified miRNA among the total mapped miRNAs. (**C**) Correlation between the read counts from the two biological replications. (**D**) Correlation between the read counts and qPCR values of 3 selected miRNAs. The abundances of the selected miRNAs as determined by qPCR were expressed by the 35-Ct miRNA.

Because the abundance of most miRNAs was relatively low in the serum exosomes, in the present study, the detectable miRNAs were defined as those that had at least one read per million-mapped miRNA reads (TPM, tags per million). This threshold excluded those miRNAs with only one read in both samples. Hence, 427 and 448 known miRNAs were identified, among which 409 were common between samples A and B, and 18 and 39 were unique to samples A and B, respectively (Figure 4A). In total, 466 miRNAs were identified (Supplementary Table 2), which corresponded to approximately 25% known miRNAs (1915 mature miRNAs in the miRBase, Mus musculus). As shown in Figure 3A and 3B, almost all of these miRNAs were 21–23 nt in length, with the predominant species being 22 nt (65.56 ± 7.65%). This length is typical of Dicer-processed products. The significant feature of serum exosomal miRNAs was their vast dynamic range of abundance. The read counts of these identified miRNAs ranged from 1 to 448,074 (Figure 4B, Supplementary Table 2), and the 20 and 100 most abundant miRNAs accounted for approximately 86% and 99% of all the mapped reads, respectively (Supplementary Table 2). The remaining 366 miRNAs accounted for less than 1.5% (Figure 4B). These listed miRNAs were the large proportion of detectable miRNAs and represented a repertoire of miRNAs contained in the mSEs.

To determine the reproducibility of biological replicates, linear regression analyses were performed on the log2-transformed read counts derived from two independent RNAseq experiments. The Pearson correlation coefficient between samples A and B was 0.9682 (Figure 4C). Three miRNAs with different abundances were selected for qPCR to evaluate the quantitative accuracy of the RNA sequencing data. The average read counts of miR-192-5p, miR-351-5p and miR-802-5p were 14,281, 219 and 12, respectively, and representing the high, middle and low abundance levels for the identified miRNAs (Supplementary Table 2). The quantitative PCR results revealed that the relatively expression level of miR-192-5p was 25- and 200-fold higher than that of miR-351-5p and miR-802-5p, respectively (Figure 4D). The Pearson correlation coefficient between the RNA sequencing and qPCR data was 0.9954. These results demonstrated the good reproducibility of the mSEs isolation, exosomal RNA extraction, library preparation and RNAseq and that the sequencing data could accurately reflect the expression levels of the identified miRNAs. However, considering the comparably small sample size of this study, the total variability of mSEs miRNAs could be more diverse.

### Cell and tissue sources of mSEs

Recent studies have analyzed the expression patterns of miRNAs in blood cells [9, 17–19] and a variety of tissues [20–23]. These studies demonstrated that some miRNAs are predominantly or even particularly expressed in certain tissues and cells, and corresponding databases have been established based on these data [22, 24]. Given the obtained mouse serum exosomal miRNA profile and existing knowledge regarding cell and/or tissue specific miRNAs, we proposed the possible sources of the mSEs. As indicated in Table 2, among the top 20 miRNAs, miR-486, miR-16, miR-451 and miR-92a are mainly expressed by RBCs [9, 18] and account for 45% reads of the mapped miRNAs. MiR-192 is one of the most abundant miRNAs in the liver [25], and miR-143 is highly expressed in smooth muscle cells (SMCs), fibroblasts [26] and intestinal and lung cells [24]. In addition to those high-level miRNAs, many miRNAs from other cell components of the blood were among the top 100, such as miR-223-3p, miR-146a-5p, miR-150-5p, myeloid cell specific miRNAs [9], and miR-484, which was the most abundantly expressed miRNA in platelets [19] (Supplementary Table 2). In addition, a panel of tissue-specific miRNAs was also identified, including miR-122 (liver), miR-133a (muscle) and miR-208a (heart) [27]. It worth noting that we did not find brain-specific miRNAs, such as miR-124 [20] in the serum exosomes. In short, these data suggested that under normal conditions, most mSEs were derived from the RBCs, liver, intestines, and heart. Other blood cell types, such as PBMCs and platelets, were also contributed to the mSEs, but in relatively small proportions.

**Table 2:** Twenty most abundant mouse serum exosomal miRNAs and their main origins

Target id	Sample A	Sample B	Sample A TPM	Sample B TPM	Cell or Tissue enriched
mmu-miR-486a-5p * mmu-miR-486b-5p	444299	448074	361742.49	390129.95	RBCs [18]
**mmu-miR-22-3p** *	194001	142702	157953.10	124248.06	Uncertain
**mmu-miR-16-5p** *	65817	62173	53587.35	54132.91	RBCs [18]
**mmu-miR-10b-5p**	48251	52538	39285.34	45743.89	Adipose [24]
**mmu-miR-27b-3p** *	39570	40522	32217.38	35281.77	Uncertain
**mmu-miR-10a-5p**	35193	35777	28653.68	31150.39	Uncertain
**mmu-miR-191-5p**	32193	29762	26211.12	25913.24	RBCs [24]
**mmu-miR-21a-5p** *	26485	19436	21563.74	16922.57	Liver, Intestines [21]
**mmu-miR-25-3p**	23657	22650	19261.22	19720.95	Uncertain
**mmu-miR-148a-3p**	18568	13810	15117.83	12024.12	Pancreas [21]
**mmu-miR-143-3p** *	18094	20407	14731.90	17768.01	Intestines and Lung [24]
**mmu-miR-451a**	16994	18261	13836.29	15899.52	RBC, Bone marrow [18]
**mmu-miR-192-5p**	15621	12941	12718.42	11267.50	Intestines, Liver and Kidney [21, 22, 24]
**mmu-miR-181a-5p** *	15107	16715	12299.92	14553.45	PBMC [24, 53]
**mmu-miR-30a-5p** *	14533	9336	11832.58	8128.69	Kidney and Lung [24]
**mmu-miR-126b-3p** *	12216	12261	9946.11	10675.43	Endothelial cells [54]
**mmu-miR-92a-3p** *	10236	10295	8334.02	8963.67	RBC, Muscle
**mmu-let-7i-5p** *	8558	8231	6967.81	7166.58	PBMC [55]
**mmu-miR-486a-3p**	8209	8420	6683.66	7331.14	RBC [18]
**mmu-miR-128-3p**	8055	5748	6558.28	5004.68	Uncertain

*miRNAs involved in immune and inflammatory responses.

### Predicted novel miRNAs

**Figure 5:**
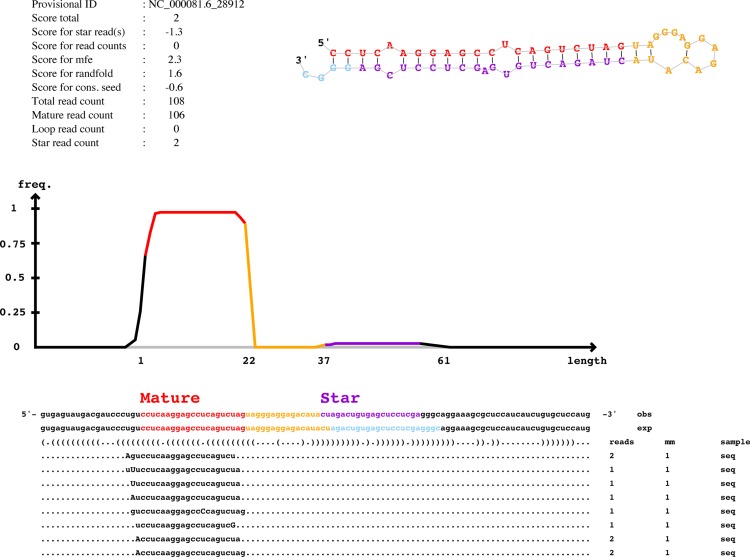
Predicted novel miRNAs in mSEs The graphic depicts the representative novel miRNA as predicted by miRDeep2. mm, number of mismatches, indicated by uppercase letters. Information regarding the miRDeep2 scores is provided in the upper left, whereas the upper right illustrates the secondary structure of the novel miRNA as predicted by Randfold software.

In the over 25 million reads obtained from small RNAseq, only five novel miRNAs were identified (Supplementary Table 3). Compared with these abundant serum exosomal miRNAs, the reads of these five were relatively few. Figure 5 shows the predicted miRNA with the greatest number of mature reads (108 reads) and its secondary structure as predicted by Randfold software. As an additional point, this result suggested that the current miRBase covers almost all of the serum exosomal miRNA genes.

### Bioinformatics analysis of the top 20 miRNAs

To analyze the potential biological functions of mSEs, the targets of the top 20 miRNAs, which accounted for 86% of total mapped reads, were predicted using the microRNA target filter in the IPA software. With the selection of ingenuity expert findings and experimentally observed, 118 targets of the top 20 miRNAs were identified (Supplementary Table 4). Core analysis revealed that the upstream regulators of these 118 targets were TGFβ1, TP53 and estradiol, and the molecular and cellular functions of these targets were mainly involved in cell growth and proliferation. Then biological functions of these 20 miRNAs were further analyzed using the IPA software. Perhaps owing to the fact that most of the existing studies on miRNAs are focused on tumors, except for a category related to inflammatory response, all of the other miRNAs in the top 10 were related to carcinoma or tumors (Supplementary Table 5). This was consistent with the results provided by core analysis, 14 of the top 20 miRNAs (labeled with * in Table 2) were within the Inflammatory Response category, including well-known immune miRNAs, such as miR-181 [28], miR-21[29] and let-7i [30]. Therefore, by delivering their miRNAs content, serum exosomes could be involved in modulating cell proliferation and inflammatory responses.

### Serum exosomes primed macrophage polarization towards the M2 phenotype

Macrophages are essential components of innate and acquired immunity and play central roles in inflammation and host defense [31, 32]. Plasticity and flexibility are two key features of macrophages and of their activation states. The M1- and M2-type macrophage responses, abbreviated as M1/M2, describe the two major and opposing activities of macrophages: M1 activity inhibits cell proliferation and causes tissue damage, whereas M2 activity promotes cell proliferation and tissue repair [32]. To test the hypothesis prompted by the bioinformatics analysis, we examined the effects of mSEs on the phenotype of the frequently used mouse macrophage cell line RAW264.7. First, the transfection efficiency of the mSEs on RAW 264.7 cells was evaluated, as this is the fundamental to their biological functions. As described in methods, mSEs labeled with SYTO were added to RAW 264.7 cells, and two hours later, almost all of the cells were stained green (Figure 5A), which demonstrated the high efficiency of mSEs in transferring their RNA cargos to macrophages.

Whole serum, exosomes-depleted serum, or serum exosomes suspended in an equal volume of media were then added to RAW264.7 cells. After 12 hours, a panel of cytokines, including 4 M1 markers and 3 M2 markers, was detected by qPCR. Whole serum treatment significantly increased the expression of all the three M2 markers, Arg1, IL-10 and TGF-β1, whereas expression of the M1 markers, iNOS, IL 1β and TNF-α, but not IL-6, were significantly suppressed. Notably, levels of Arg1 and iNOS, two representative markers of the M1 and M2 phenotypes [33], were 2,000-fold higher and 25-fold lower than the control, respectively. These data manifested that the macrophages were polarized towards the M2 phenotype. Most interestingly, the serum exosomes had nearly the same effect as did the whole serum, and in most cases, the effects on cytokine expression were greater than those induced by the exosomes-depleted serum, especially for Arg1 and iNOS (Figure 5B). This result indicated that serum exosomes, in addition to circulating hormones and cytokines, could play important roles in intercellular communication and contributed to homeostasis.

## DISCUSSION

In the present study, we found that tRNA fragments and miRNAs were the two predominant small RNA species of mSEs and accounted for approximately 60% and 10% of mapped reads, respectively. Moreover, based on existing knowledge of cell- and tissue-specific miRNAs, our data suggested that the mSEs were mainly derived from RBCs, liver and intestines. Bioinformatics analysis revealed that more than half of the top 20 mouse serum exosomal miRNAs were involved in inflammatory responses, and further *in vitro* experiments demonstrated that mSEs could prime macrophages towards a M2 phenotype. To the best of our knowledge, this is the first systemic and functional study of mSEs. In addition to providing a reference for future functional and biomarker studies on serum exosomes, our data also suggested their potential roles in maintaining homeostasis of the internal environment under normal physiological conditions.

### MiroRNAs and tRNAs are the two predominant small RNA contents of mSEs

The existing RNAseq studies on the small RNA contents of serum exosomes are currently confined to humans [13, 15]. As with human serum exosomes, our RNAseq data showed that mSEs also contained a variety of small RNA species, and a few miRNAs accounted for most of the exosomal miRNAs identified (Figure 3). Unexpectedly, we noticed a significant difference between human and mouse samples in their proportions of RNA species. MiRNAs are considered as the major functional molecules of exosomes in intercellular communication and are intensively studied among the exosomal RNA contents [10]. Our data showed that miRNAs accounted for only approximately 10% of the mappable reads (Figure 3C and 3D). However, a study using the same small RNA library preparation kits and sequencing platform found that more than 70% of the mappable reads in human plasma exosomes were miRNAs [15], and another study using the Ion Torrent PGM platform also showed that over 40% of the small RNAs found in human serum exosomes were miRNAs [34]. These data suggested that miRNAs were the main small RNA components of human plasma exosomes. In contrast, our data showed that tRNA fragments were the most abundant small RNA components in mSEs (Figure 3) and accounted for over 60% of mapped reads. Why is there such a significant difference in the small RNA species between different species?

Several explanations for this discrepancy could exist, such as different methods for exosomes preparation and RNA extraction, different small RNAseq library building methods, and different sequencing platforms and data processing approaches, but we found that the different length distributions of the exosomal RNA could be the main reason. The lengths of the mouse serum exosomal RNAs ranged from 15 to 200 nt, and the small RNAs (15–40 nt) were approximately 30% of the total exosomal RNA (Figure 2E and 2F). However, the dominant size of the human serum exosomal RNA had lengths between 18–28 nt [15]. As illustrated in Figure 3, the mapped miRNAs had lengths between 18–28 nt. This length distribution pattern not only elucidated the reason for the high proportion of miRNAs but also helped to explain the lack of tRNA fragments in human serum exosomes, since their small RNAs of longer than 30 nt were very few. Mouse animal models are widely used in basic and translational medicine researches; therefore, it is necessary to further confirm these differences among different species and to determine whether these differences are implicated in the different strategies of exosomes packaging.

Another point worth noting is these detected tRNAs were tRNA fragments. The length that can be detected by the small RNAseq is less than 40 nt, whereas the lengths of mature tRNAs are between 70 and 90 nt. Many experiments have shown that the exosomal RNA component is very stable [35]; therefore, these RNA fragments should be derived from their host cells rather than digested after packaging into exosomes. As serum exosomes contain large amounts of tRNA and rRNA fragments, they could also act as a mechanism for disposal of unwanted RNA contents, similar to the roles of exosomes in disposal transferrin receptor during the maturation of erythrocytes [2]. For this reason, when exploring the functions of exosomes in intercellular communication, their roles in the disposal of those unwanted RNA components should not be ignored.

### Origins of mSEs

Tissue- and/or cell-specific expression of miRNAs is fundamental to biomarker studies, but to date, the contributions of different tissues or cell types to serum exosomes remains unclear. The only such efforts to understand these questions were implemented by Hunter et al., who used flow cytometry and cell-specific surface markers. These authors found that the majority of the peripheral blood microvesicles (MVs) are platelet-derived [19]. Our data suggested that RBCs were the main sources of serum exosomes, as 45% of the top 20 exosomal miRNAs were derived from RBCs. Recent studies based on PCR array have also claimed that blood cells are a major contributor to circulating miRNAs, and that perturbations in blood cell counts or hemolysis can greatly alter their levels [9, 18]. The discrepancy between our study and Hunter's study is mainly due to the different types of particles studied. The particles investigated by Hunter et al., were MVs with diameters of about 2ｕ m, rather than the nanoscale exosomes investigated in the present study, since the conventional FACS used in Hunter's study has limited nanoparticle detection [19].

Furthermore, for the first time, our data demonstrated that exosomes are released into the body fluid by almost all types of tissues and blood cells, such as liver, muscle, intestine, heart and myeloid cells (Table 2). Elevation or alteration of these tissue- or cell- specific exosomal miRNAs can be induced by tissue damage, and their levels can reflect the degree of damage. For instance, miRNA-122, which is abundant in hepatocytes, robustly increased in plasma exosomes following drug-induced liver injury, and miR-122 levels correlated with the degree of hepatocyte injury [36]. In our recent study, the brain-specific miR-9 and -124 significantly increased in the serum exosomes from stroke patients [37]. As illustrated in Figure 4B, most tissue-specific exosomal miRNAs are rare. Compared with these abundant miRNAs in the top 100, the other 366 miRNAs accounted for less than 1.5 % of the total exosomal miRNAs. This expression pattern presents a significant challenge for biomarker studies on tissue-specific exosomal miRNAs and exosomes per se, especially in screening for exosomal protein biomarkers. Thus, to isolate cell- or tissue- specific exosomes from whole serum exosomes will greatly improve the sensitivity and accuracy, and can avoid those off-target mistakes [9]. Emerging micro- and nanotechnologies have already been tested to isolate and detect exosomes in clinical samples [38]. Finally, considering most of these specific miRNAs are currently determined at the tissue level without considering their multiple different cell types of origin, clarifying the cellular sources of these serum exosomes will greatly promote the biomarkers studies as theoretically, each of them should possess a unique miRNA expression pattern.

### Roles of serum exosomes in modulating inflammatory responses

**Figure 6:**
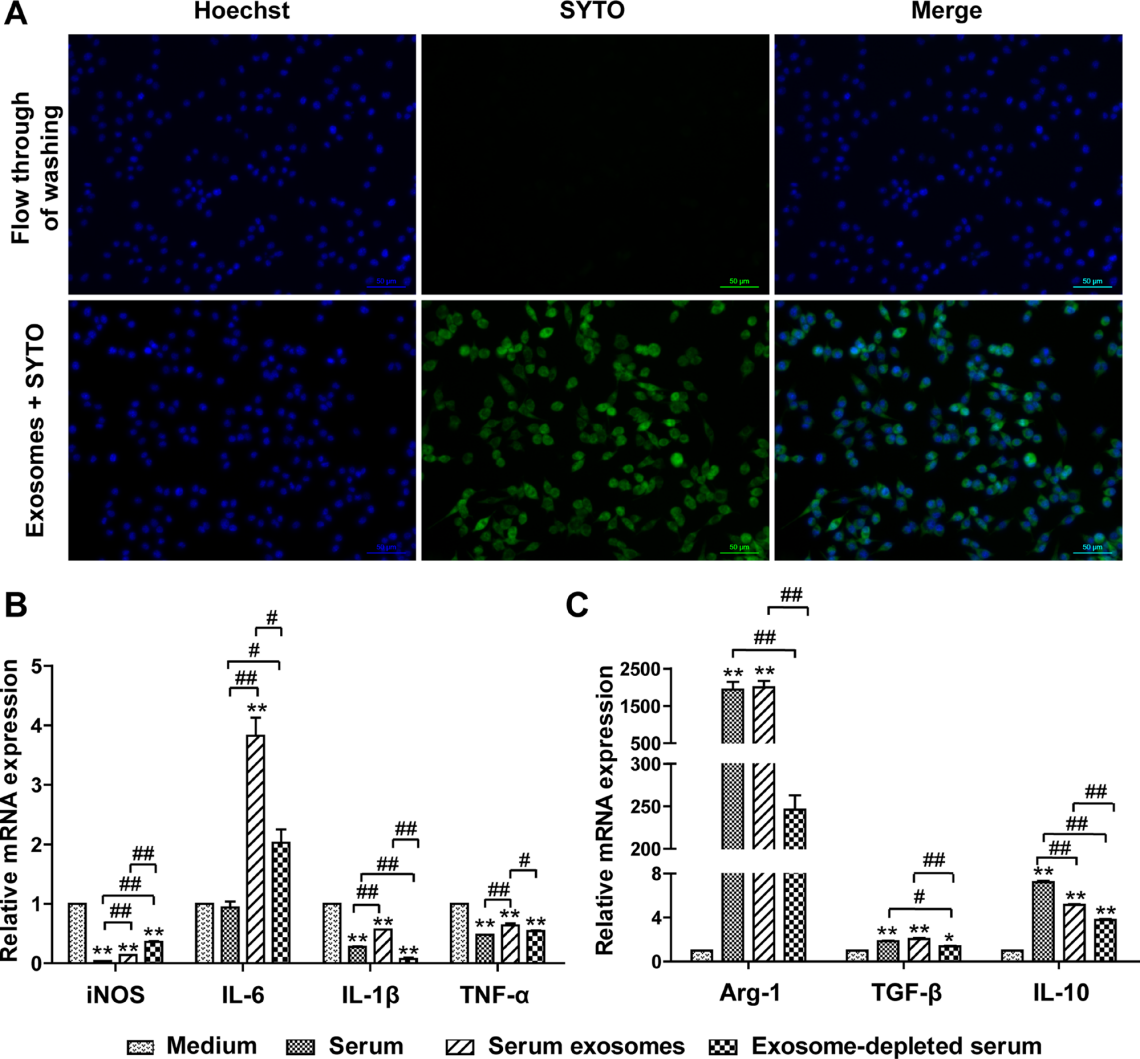
Uptake of mSEs by RAW264.7 cells and the effects of mSEs on M1/M2 gene expression of RAW264.7 cells (**A**) Fluorescence microscopy images of RAW264.7 cells incubated with SYTO-labeled serum exosomes or 3rd flow through of SYTO labeling. Live cells are stained with Hoechst stain to detect nuclei (blue). Bar = 50 ｕm. The Images are representative of 3 independent experiments. (**B**) The effects of serum, serum exosomes and exosomes-depleted serum on the transcript levels of M1 (B) and M2 (**C**) cytokines. *P < 0.05, **P < 0.01 vs control group; ^#^*P* < 0.05, ^##^*P* < 0.01 between the indicated groups.

The most interesting finding of this study was that serum exosomes from normal mice primed the macrophages towards the M2 phenotype (Figure 6). The contribution of exosomes from both immune and nonimmune cells to immune regulation has been well reported. For example, exosomes released from activated human T cells participate in IL-2-mediated immune responses [39], whereas MSC-derived exosomes improve ischemic heart function by restraining the inflammatory response and stimulating neovascularization [12]. Recent studies have also investigated the roles of circulating exosomes in mediating immunological responses, such as serum-derived exosomes from antigen-fed mice prevented allergic sensitization in an allergic asthma model [40] and blockade of exosomes generation in sepsis dampened the sepsis-triggered inflammatory response and improved cardiac function and survival [41]. Consistent with our results, exosomes from fetal bovine serum dampened the response of primary macrophages to lipopolysaccharide (LPS) challenge [42]. However, the molecular mechanism underlying this suppression under normal conditions is unclear.

MiRNAs are considered to be the main functional components of exosomes [10], and accumulating evidence suggests a role for specific miRNAs in modulating inflammatory response [43]. Our data showed that more than half of the top 20 exosomal miRNAs were involved in inflammatory responses, and some of them have been reported to inhibit innate immunity by negative regulation of TLR-4 signaling or down regulation of inflammatory signaling pathways [44, 45]. Transferring these miRNAs will certainly change the gene expression within the macrophages, and these abundant miRNAs should be at least partially responsible for the effects of serum exosomes on macrophage polarization. In addition to these abundant miRNAs, those with low abundances could also participate in these processes. MiR-155 and miR-146a are two critical immunoregulatory miRNAs involved in modulating inflammation and macrophage polarization [43]. A recent study by Alexander demonstrated that miR-155 and miR-146a were present in exosomes and could be passed between immune cells [46]. Moreover, exosomal miR-146a inhibited, whereas miR-155 promoted, endotoxin-induced inflammation in mice [46]. Our results also showed the existence of both miR-155 and miR-146a in mSEs, and intriguingly, the amount of miR-146a-5p was 200-fold higher than that of miR-155-5p (Supplementary Table 2). This expression pattern also indicated the anti-inflammatory tendency of serum exosomes derived from normal mice as well.

An unresolved puzzle for exosomes studies is whether the miRNA content or a specific miRNA in the exosomes is sufficient to exert effective biological functions, even though the accumulating studies have reported that the biological functions of exosomes could be mediated by even a single type of miRNA [47, 48]. As shown by our study and others, miRNAs account for a small part of the exosomal small RNAs that ranged from 15–40 nt, and these small RNAs are only a small portion of the total exosomal RNA (Figure 3). Considering the limited amount of exosomal RNA, the numbers of a specific miRNAs should be much lower. In a recent study, Chevillet et al. quantified the number of a specific miRNA per exosomes using NanoSight and a real-time PCR-based absolute quantification method [49]. They found that on average, there was far less than one molecule of a given miRNA per exosome [49]. Although the biological functions of serum exosomes can be explained by their highly efficient transfection, specific binding, constant release and relatively high concentration of miRNAs in serum exosomes, we still cannot overlook the potential biological effects of the remaining exosomal content, including the exosomal RNA species other than miRNAs, and their protein and lipid compositions. As demonstrated by our study, tRNA fragments are the predominant small RNA species in mSEs (Figure 3). A recent study demonstrated that endogenous tRNA-derived fragments suppressed breast cancer progression via YBX1 displacement [50], Their study suggests that transfer RNA fragments are not random by-products of tRNA degradation or biogenesis, but an abundant and novel class of short RNAs with precise sequence structures that have specific expression patterns and biological roles [50]. Furthermore, due to the length limitation of the constructed small RNA library, almost all of the existing studies on serum exosomal RNA content are focused on the RNA species under 40nt, and our knowledge on the exosomal RNA contents within the length of 40–150 nt is still lacking. From the perspective of exosomal RNA content, RNAs of this size are the main components. Proteins are another main exosomal component, and a recent proteomic profiling of dextran sulfate sodium-induced acute ulcerative colitis mice serum exosomes identified a panel of exosomal proteins potentially involved in macrophage activation [51]. Thus, unlike the information conferred by single type of miRNA or protein, serum exosomes as well as exosomes derived from other sources should be considered as a package of information released from their host cells, and their biological functions are the net effects of these exosomal RNAs, proteins and other components.

Taken together, this study established a reference for biomarker studies of mouse serum exosomes, proposed the likelihood of their origin, and provided the first evidence that serum exosomes could prime the macrophage towards an M2 phenotype under normal conditions. Therefore, besides hormones and cytokines, serum exosomes can contribute to the homeostasis via their roles in the intercellular communications. To comprehensively understand the molecular mechanism underlying their multiple biological functions, further studies are needed to profile the other contents in the serum exosomes, especially other RNA species and proteins.

## MATERIALS AND METHODS

### Animals and serum preparation

The experimental design and procedures involving animals and their care were conducted according to the National Institutes of Health Guide for Care and Use of Laboratory Animals and were approved by the Animal Experimental committee of Jinan University.

Male C57BL/6 mice (10 weeks old) were purchased from Medical Experimental Animal Center of Guangdong province. Anesthesia was induced with 1% chloral hydrate. After collecting by left ventricular puncture, blood was placed at room temperature (RT) or 37°C for 1 h to allow it to clot. Afterwards, samples were centrifuged at 500 g at 4°C for 10 min, the clear serum was amber and those with obvious hemolysis (pink or faint red) were discarded at this stage. Serum from 5 to 8 mice was then pooled and centrifuged at 500 g for 10 min and followed by another 10,000 g for 20 min at 4°C, the supernatant was immediately aliquoted and stored at −80°C.

### Cell culture

RAW264.7 cells, a murine macrophage cell line, were obtained from the American Type Culture Collection (ATCC, American Type Culture Collection, Manassas, VA, USA) and cultured in high glucose DMEM medium containing 10% heat-inactivated fetal bovine serum and antibiotics (100 U/mL penicillin and 100 U/mL streptomycin) (Gibco Laboratories, Carlsbad, CA, USA). Cells were incubated at 37°C in a humidified atmosphere of 5% CO2 in air and the medium was changed every 2–3 days. The cultures were passaged when they reached 80% confluence.

### Serum exosomes isolation

An ExoQuick kit (System Biosciences, Mountain View, CA) was used to isolate mSEs per the manufacturer's instructions with minor modifications. Briefly, frozen mouse serum was thawed on ice and centrifuged at 21,000 g for 15 minutes at 4°C to remove debris. The supernatant was then transferred to a new tube, and 1/4 volume EXOQ5A-1 solution was added and mixed gently. After incubation at 4°C for 2 h, the mixture was centrifugation at 1,500 g for 30 minutes, and the supernatant was removed by aspiration. After another 5 min centrifugation to remove the residual supernatant, pellet-containing exosomes was re-suspended in nuclease-free water or corresponding buffer for RNA or protein extraction.

### Nanoparticle tracking analysis (NTA)

Size distribution and concentration of the isolated mSEs were measured by NTA as described previously [37]. For calibration, 100 nm polystyrene latex standards and particle-free PBS were tested routinely. Before measurement, the isolated exosomes were homogenized and 1,000-fold diluted in particle-free PBS. The measurement time was 60 s and the Frames Per Second was 25. All samples were tested three times.

### Transmission electron microscopy (TEM)

For morphology observation, electron microscopy samples of mSEs were prepared as described by Thery et al. [52]. Exosomes suspended in PBS was first mixed with equal volume of 4% paraformaldehyde. Then, 10 μl the fixed samples were transferred onto Formvar/carbon-coated nickel grids and the membranes were allowed to adsorb for 20 min at RT. After wash and fixation with 50 μl 1% w/v glutaraldehyde, samples were contrasted with 4% w/v Uranyl Acetate (UA) for 5 min and embedded in a mixture of 4% UA and 2% Methyl cellulose (100:900) solution for 10 min on ice. The excess fluid was blotted and air-dried the grid. The morphology and size of exosome were examined using a TEM (JEM-2100 JEOL, Tokyo, Japan).

### Western blotting analysis

Exosomal protein was extracted with RIPA lysis buffer (Pierce, Rockford, IL). The protein concentration was determined by BCA assay (Abcam, USA). Forty-microgram protein was separated by 12% sodium dodecyl sulfate-polyacrylamide gel electrophoresis and electroblotted onto a PVDF membrane (Millipore, USA). The membrane was blocked with 5% bovine serum albumin for 1 h at RT, and then incubated overnight with antibodies against CD63 (System Biosciences, Mountain View, CA, USA) and TSG101 (Abcam, Cambridge, MA, USA). After 3 times wash and incubation with HRP-conjugated secondary antibodies, the proteins were detected by enhanced chemiluminescence (Pierce, Rockford, IL, USA).

### Exosomal RNA extraction and quantitation

Total RNA was extracted from the serum exosomes using SeraMir kit (System Biosciences, Mountain View, CA, USA) according to the manufacturer's protocol. Purity and quantity of the isolated exosomal RNA were determined using Nano Drop (Thermo Fisher Scientific, Wilmington, DE). Integrity and size distribution of exosomal RNA were assessed by using Agilent Bioanalyzer 2100 system combined with RNA 6000 pico kit and small RNA kit (Agilent Technologies, Palo Alto, CA, USA).

### Small RNA library construction and deep sequencing

The NEB Next^®^ Small RNA Library Prep Set for Illumina^®^ (NEB, Ipswich, MA, USA) was used to generate exosomal small RNA libraries for next-generation sequencing on the Illumina platform. Per manufacturer's recommendations, total exosomal RNA was subjected to sequential 3′ and 5′ adapter ligations followed by reverse-transcription into a cDNA library. After PCR enrichment, the miRNA library was size (140 bp) selected from an 8% PAGE-gel and the library quality was assessed on the Agilent Bioanalyzer 2100 system (Agilent Technologies, Palo Alto, CA, USA). The qualified cDNA libraries were used for cluster generation and sequenced on the Illumina HiSeq 2500 platform (Illumina, San Diego, CA, USA) to obtain 125 bp single-end reads.

### Sequencing data analysis

A flow chart describing the sequencing data analysis is shown in Figure 1. First, by using cutadapt (version 1.7.1) and the Fastx toolkit (version 0.0.13), the adaptors as well as low-quality reads (Q30 < 80%) and low-complexity reads were removed from the raw reads (the total unfiltered reads obtained from the Hiseq 2500), and the remaining reads were designated as clean reads. Afterwards, the reads shorter than 15 nt or longer than 40 nt were filtered from these clean reads to obtain the valid reads. The valid reads were first aligned against a reference mouse genome (GRCm38) using Bowtie tools. Then, the valid reads were aligned against Rfam (version 11.0) using Blastn software to annotate the rRNA, snRNA, snoRNA and tRNA species. The reads filtered by Rfam were aligned against the mouse transcriptome and repeat database to identify degraded mRNA fragments and repeats. To annotate the known miRNAs, these filtered reads were blasted against mouse miRNA sequences downloaded from miRBase (Release 21) using Blastn software. Finally, the unannotated reads were used to predict novel miRNAs using MiRDeep 2 software (Version 2.0.0.8). The length distribution and unique reads were generated from the obtained valid reads and mapped reads.

### Quantitative real-time PCR (qPCR) for miRNA and RNA

The exosomal miRNAs were detected by qRT-PCR as described previously [37]. MiDETECT A Track™ miRNA qRT-PCR kits containing miRNA-specific forward primers and sequence complementary to the poly (T) adapter as the reverse primer were purchased from Riobio (Riobio Inc, Guangzhou, China). According to the manufacturer's instructions, total exosomal RNA from 250 ul mouse serum was polyadenylated and reverse-transcribed into cDNA using a poly (T) adapter. Mature miRNAs expression was detected using the SYBR^®^ Premix Ex Taq™ II, Perfect Real Time kit (TaKaRa, Dalian, China) on a CFX96 PCR system (Bio-Rad; Hercules, CA, USA). The relative expression of the detected miRNAs was expressed as ΔCT = 35- mean CTmiRNA.

Total RNA was extracted from RAW264.7 cells using Trizol (Life Technologies, Carlsbad, CA). RNA quantity and quality were determined by a NanoDrop 1000 spectrophotometer (Thermo Scientific, Wilmington, DE, USA). One microgram of total RNA was used to synthesize the cDNA via the Superscript First-Strand Synthesis System for RT-PCR (Life Technologies, Carlsbad, CA). Primer sequences used for subsequent QPCR analysis were provided in Supplementary Table 1. The mRNA levels were normalized against β-actin expression and the relative expression level of each gene was presented as 2^−ΔΔCt^.

### Bioinformatics analysis

The prediction of targets for exosomal miRNAs and following molecular and cellular functional analyses of these predicted targets were carried out by using the Ingenuity Pathway Analysis (IPA) software (Ingenuity Systems, Redwood City, CA).

### Cellular uptake of mSEs

Isolated mSEs were labeled with 10 μM of nucleic acids specific dye SYTO (Life Technologies, Carlsbad, CA) for 20 min at 37°C, and washed three times with 100 kDa MWCO UltraCentrifugal Filters (Millipore, Bedford, MA) to remove the unincorporated dye. The labeled exosomes were suspended in 200 μl PBS and added immediately to RAW264.7 cells; meanwhile, the flow through from the last wash was added as a control. After 2 h of incubation at 37°C, the cellular nuclei were stained with Hoechst 33258 (8 μg/ml) for 5 min, and exosomes uptake was visualized by fluorescence microscopy (Carl Zeiss, Oberkochen, Germany).

### Statistical analysis

The data are presented as the mean ± SEM. Comparisons between means were assessed by unpaired Student's *t* test or one-way analysis of variance using SPSS10.0 software. *P* values of less than 0.05 or 0.01 were regarded as statistically significant. Unless otherwise specified, all assays were performed in triplicate.

## SUPPLEMENTARY FIGURE AND TABLES








