# Supplementary material for: Characterization of mouse serum exosomal small RNA content: The origins and their roles in modulating inflammatory response
Source: Oncotarget. 2017 Apr 27;8(26):42712–27. doi: 10.18632/oncotarget.17448 (PMC5522100; doi:10.18632/oncotarget.17448)
Supplement: Supplementary file 1 [file oncotarget-08-42712-s001.pdf]

Characterization of mouse serum exosomal small RNA content: The origins and their roles in modulating inflammatory response

Supplementary Materials

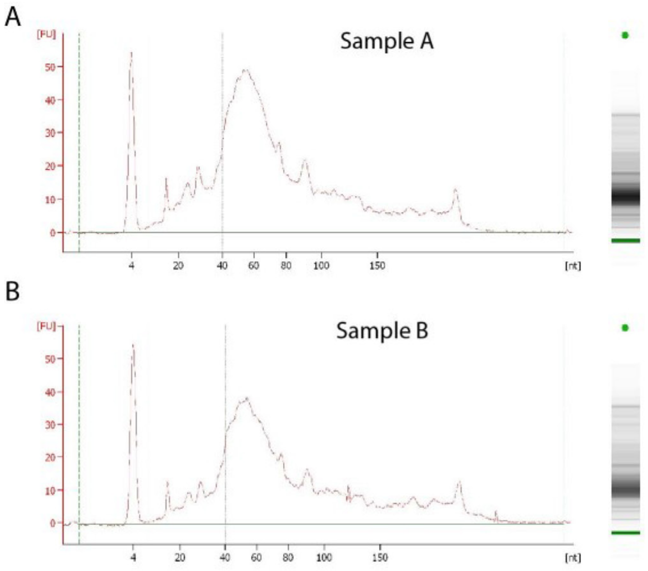

C

	Sample A	Sample B
Small RNA Concentration [pg/µl]: (0-315nt)	4,204.60	3,357.90
miRNA Concentration [pg/µl]: (0-40nt)	1,333.70	917.6
miRNA / Small RNA Ratio [%]:	32	27

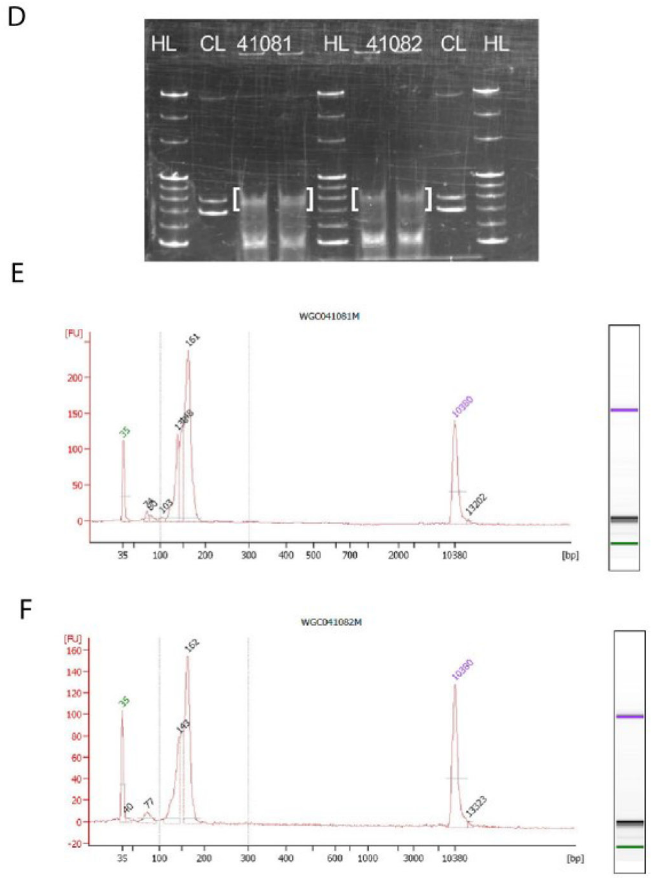

Supplementary Figure 1: Small RNA sequencing libraries construction for mSEs. The length distribution and concentration of exosomal RNA from two batches of mouse serum were examined by using Bioanalyzer. As showed in Supplementary Figure 1 (**A** and **B**), there were no obvious difference between these two samples. The quantification of serum exosomal small RNA and miRNA and the miRNA/small RNA ratio (**C**) Two small RNA libraries were constructed by using 2 ng total RNA as described in methods. After ligation and PCR amplification, the products were separated on a gel and the targeted fragments (146 bp bands) were recovered (**D**). The obtained libraries were further analyzed by Bioanalyzer and we could find the peaks at 146 bp (**E** and **F**).

Supplementary Table 1: The sequences of primers used for amplification of M1/M2 cytokines

	Gene		Primer sequence
M1	TNFα	Forward primer:	ATGGCCTCCCTCTCATCAGT
		Reverse primer:	TGGTTTGCTACGACGTGGG
	IL1-β	Forward primer:	TGCCACCTTTTGACAGTGATG
		Reverse primer:	AAGGTCCACGGGAAAGACAC
	iNOS	Forward primer:	GGTGAAGGGACTGAGCTGTT
		Reverse primer:	ACGTTCTCCGTTCTCTTGCAG
	IL-6	Forward primer:	GCCTTCTTGGGACTGATGCT
		Reverse primer:	TGCCATTGCACAACTCTTTTCT
M2	IL-10	Forward primer:	CCAAGCCTTATCGGAAATGA
		Reverse primer:	TTTTCACAGGGGAGAAATCG
	TGF-β	Forward primer:	TGCGCTTGCAGAGATTAAAA
		Reverse primer:	CGTCAAAAGACAGCCACTCA
	Arg-1	Forward primer:	TTTTAGGGTTACGGCCGGTG
		Reverse primer:	CCTCGAGGCTGTCCTTTTGA

Supplementary Table 2: List of 466 known miRNAs. See Supplementary_Table_2

Supplementary Table 3: List of novel miRNAs

Novel miRNAs predicted by miRDeep2

provisional id	miRDeep2 score	significant randfold *p*-value	total read count	mature read count	loop read count	star read count	consensus mature sequence	consensus star sequence
NC_000079.6_26381	2.3	yes	16	16	0	0	cgccgcgcgcgcccagcccc	ggacgcgggccugcagguguc
NC_000081.6_28912	2	yes	108	106	0	2	ccucaaggagccucagucuag	cuagacugugagcuccucga
NC_000075.6_19627	1.6	yes	15	15	0	0	cuauccuggauuacuugaa	caaguaaccaagaauaggc
NC_000080.6_27962	0.5	yes	19	19	0	0	gagcggucgcgccauggg	cgccggagcgccagaugn
NC_000072.6_12896	0.4	yes	2	2	0	0	cggugcgguggcggacgau	cucuggcgacacccggacggcc
NC_000075.6_18543	0.2	yes	1	1	0	0	ggcccccuggccuccuccgcg	cgaggugggaggcgccgg
NC_000069.6_6356	0.2	yes	12	12	0	0	cuagccuacagcacccgg	caauguuguguuagaa
NC_000074.6_17720	0.1	yes	1	1	0	0	uggcucaggugugguccuuu	agggucaccggaaccuggccagc
NC_000085.6_34428	0.1	yes	1	1	0	0	guuggggaaggggugaaggagaa	cucuuucuggucagguccuggggg
NC_000079.6_26661	0.1	yes	1	1	0	0	cagcccugccuucuccuc	ugcagaagcggcggcugcu
NC_000067.6_504	0	yes	1	1	0	0	gggggcagggccuggcgguggga	aucacguccgguccuucccauccccccu

Supplementary Table 4: List of 118 targets of the top 20 miRNAs. See Supplementary_Table_4

Supplementary Table 5: Diease and biological functions of the top 20 miRNAs. See Supplementary_Table_5
