# Supplementary material for: Characterization of mouse serum exosomal small RNA content: The origins and their roles in modulating inflammatory response
Source: Oncotarget. 2017 Apr 27;8(26):42712–27. doi: 10.18632/oncotarget.17448 (PMC5522100; doi:10.18632/oncotarget.17448)
Supplement: Supplementary file 2 [file oncotarget-08-42712-s002.docx]

|  | **Target ID** | **Sample A** | **Sample B** | **Sample A TPM** | **Sample B TPM** |
| --- | --- | --- | --- | --- | --- |
| 1 | mmu-miR-486a-5p>mmu-miR-486b-5p | 444299 | 448074 | 361742.49 | 390129.95 |
| 2 | mmu-miR-22-3p | 194001 | 142702 | 157953.10 | 124248.06 |
| 3 | mmu-miR-16-5p | 65817 | 62173 | 53587.35 | 54132.91 |
| 4 | mmu-miR-10b-5p | 48251 | 52538 | 39285.34 | 45743.89 |
| 5 | mmu-miR-27b-3p | 39570 | 40522 | 32217.38 | 35281.77 |
| 6 | mmu-miR-10a-5p | 35193 | 35777 | 28653.68 | 31150.39 |
| 7 | mmu-miR-191-5p | 32193 | 29762 | 26211.12 | 25913.24 |
| 8 | mmu-miR-21a-5p | 26485 | 19436 | 21563.74 | 16922.57 |
| 9 | mmu-miR-25-3p | 23657 | 22650 | 19261.22 | 19720.95 |
| 10 | mmu-miR-148a-3p | 18568 | 13810 | 15117.83 | 12024.12 |
| 11 | mmu-miR-143-3p | 18094 | 20407 | 14731.90 | 17768.01 |
| 12 | mmu-miR-451a | 16994 | 18261 | 13836.29 | 15899.52 |
| 13 | mmu-miR-192-5p | 15621 | 12941 | 12718.42 | 11267.50 |
| 14 | mmu-miR-181a-5p | 15107 | 16715 | 12299.92 | 14553.45 |
| 15 | mmu-miR-30a-5p | 14533 | 9336 | 11832.58 | 8128.69 |
| 16 | mmu-miR-126b-3p | 12216 | 12261 | 9946.11 | 10675.43 |
| 17 | mmu-miR-92a-3p | 10236 | 10295 | 8334.02 | 8963.67 |
| 18 | mmu-let-7i-5p | 8558 | 8231 | 6967.81 | 7166.58 |
| 19 | mmu-miR-486a-3p | 8209 | 8420 | 6683.66 | 7331.14 |
| 20 | mmu-miR-128-3p | 8055 | 5748 | 6558.28 | 5004.68 |
| 21 | mmu-miR-486b-3p | 7660 | 7863 | 6236.67 | 6846.17 |
| 22 | mmu-miR-93-5p | 7574 | 6683 | 6166.65 | 5818.77 |
| 23 | mmu-miR-423-5p | 7206 | 7378 | 5867.03 | 6423.89 |
| 24 | mmu-miR-15a-5p | 7177 | 7144 | 5843.42 | 6220.15 |
| 25 | mmu-miR-378a-3p | 6203 | 5478 | 5050.40 | 4769.60 |
| 26 | mmu-miR-27a-3p | 5528 | 5362 | 4500.83 | 4668.60 |
| 27 | mmu-miR-30d-5p | 5341 | 4361 | 4348.57 | 3797.04 |
| 28 | mmu-miR-199a-3p>mmu-miR-199b-3p | 4980 | 6487 | 4054.65 | 5648.11 |
| 29 | mmu-miR-103-3p | 4969 | 4338 | 4045.70 | 3777.02 |
| 30 | mmu-miR-107-3p | 4826 | 4165 | 3929.27 | 3626.39 |
| 31 | mmu-miR-29a-3p | 4539 | 3170 | 3695.60 | 2760.06 |
| 32 | mmu-miR-142a-5p | 4339 | 4330 | 3532.76 | 3770.05 |
| 33 | mmu-miR-144-3p | 4314 | 4560 | 3512.40 | 3970.31 |
| 34 | mmu-let-7f-5p | 4022 | 3423 | 3274.66 | 2980.34 |
| 35 | mmu-miR-26a-5p | 3659 | 3048 | 2979.11 | 2653.84 |
| 36 | mmu-miR-182-5p | 3448 | 830 | 2807.32 | 722.67 |
| 37 | mmu-let-7g-5p | 2980 | 2544 | 2426.28 | 2215.01 |
| 38 | mmu-miR-130a-3p | 2928 | 2405 | 2383.94 | 2093.99 |
| 39 | mmu-miR-125a-5p | 2845 | 2697 | 2316.36 | 2348.23 |
| 40 | mmu-miR-151-3p | 2718 | 2692 | 2212.96 | 2343.88 |
| 41 | mmu-miR-423-3p | 2718 | 2393 | 2212.96 | 2083.54 |
| 42 | mmu-miR-186-5p | 2428 | 2321 | 1976.85 | 2020.85 |
| 43 | mmu-let-7c-5p | 2358 | 2153 | 1919.85 | 1874.58 |
| 44 | mmu-miR-150-5p | 2323 | 2281 | 1891.36 | 1986.03 |
| 45 | mmu-miR-23a-3p | 2301 | 2235 | 1873.44 | 1945.97 |
| 46 | mmu-miR-221-3p | 2238 | 1954 | 1822.15 | 1701.31 |
| 47 | mmu-miR-30b-5p | 2077 | 1395 | 1691.07 | 1214.60 |
| 48 | mmu-let-7a-5p | 1799 | 1689 | 1464.72 | 1470.58 |
| 49 | mmu-miR-99b-5p | 1745 | 1661 | 1420.76 | 1446.20 |
| 50 | mmu-miR-375-3p | 1723 | 949 | 1402.84 | 826.28 |
| 51 | mmu-miR-215-5p | 1702 | 1634 | 1385.75 | 1422.69 |
| 52 | mmu-let-7d-3p | 1445 | 1287 | 1176.50 | 1120.57 |
| 53 | mmu-miR-484 | 1306 | 1182 | 1063.33 | 1029.15 |
| 54 | mmu-let-7d-5p | 1262 | 1146 | 1027.50 | 997.80 |
| 55 | mmu-miR-181c-5p | 1222 | 1235 | 994.94 | 1075.29 |
| 56 | mmu-miR-146a-5p | 1171 | 1321 | 953.41 | 1150.17 |
| 57 | mmu-miR-425-5p | 1170 | 1100 | 952.60 | 957.75 |
| 58 | mmu-miR-434-3p | 1148 | 645 | 934.69 | 561.59 |
| 59 | mmu-miR-106b-5p | 1144 | 931 | 931.43 | 810.60 |
| 60 | mmu-miR-144-5p | 1142 | 1077 | 929.80 | 937.72 |
| 61 | mmu-miR-223-3p | 1137 | 1068 | 925.73 | 929.89 |
| 62 | mmu-miR-130b-3p | 1102 | 995 | 897.23 | 866.33 |
| 63 | mmu-miR-181b-5p | 1034 | 1269 | 841.87 | 1104.90 |
| 64 | mmu-miR-127-3p | 932 | 616 | 758.82 | 536.34 |
| 65 | mmu-miR-152-3p | 893 | 981 | 727.07 | 854.14 |
| 66 | mmu-let-7b-5p | 892 | 856 | 726.25 | 745.30 |
| 67 | mmu-miR-320-3p | 866 | 832 | 705.09 | 724.41 |
| 68 | mmu-miR-328-3p | 854 | 676 | 695.32 | 588.58 |
| 69 | mmu-miR-301a-3p | 828 | 747 | 674.15 | 650.40 |
| 70 | mmu-miR-30e-5p | 814 | 708 | 662.75 | 616.44 |
| 71 | mmu-miR-378c | 808 | 619 | 657.86 | 538.95 |
| 72 | mmu-miR-744-5p | 717 | 604 | 583.77 | 525.89 |
| 73 | mmu-miR-203-3p | 684 | 771 | 556.90 | 671.30 |
| 74 | mmu-miR-101a-3p | 676 | 635 | 550.39 | 552.88 |
| 75 | mmu-miR-340-5p | 656 | 536 | 534.11 | 466.69 |
| 76 | mmu-miR-3074-5p | 632 | 671 | 514.57 | 584.23 |
| 77 | mmu-miR-141-3p | 611 | 540 | 497.47 | 470.17 |
| 78 | mmu-miR-92b-3p | 607 | 381 | 494.21 | 331.73 |
| 79 | mmu-miR-19b-3p | 599 | 609 | 487.70 | 530.25 |
| 80 | mmu-miR-15b-5p | 577 | 577 | 469.79 | 502.38 |
| 81 | mmu-miR-23b-3p | 574 | 619 | 467.34 | 538.95 |
| 82 | mmu-miR-1839-5p | 556 | 503 | 452.69 | 437.95 |
| 83 | mmu-miR-30c-5p | 556 | 396 | 452.69 | 344.79 |
| 84 | mmu-miR-126a-3p | 551 | 564 | 448.62 | 491.06 |
| 85 | mmu-miR-1198-5p | 529 | 549 | 430.70 | 478.00 |
| 86 | mmu-miR-106b-3p | 521 | 535 | 424.19 | 465.81 |
| 87 | mmu-miR-574-3p | 473 | 542 | 385.11 | 471.91 |
| 88 | mmu-miR-497a-5p | 469 | 558 | 381.85 | 485.84 |
| 89 | mmu-miR-24-2-5p | 456 | 426 | 371.27 | 370.91 |
| 90 | mmu-miR-532-5p | 433 | 454 | 352.54 | 395.29 |
| 91 | mmu-miR-140-3p | 426 | 533 | 346.84 | 464.07 |
| 92 | mmu-miR-149-5p | 420 | 408 | 341.96 | 355.24 |
| 93 | mmu-miR-676-3p | 416 | 417 | 338.70 | 363.07 |
| 94 | mmu-miR-125b-5p | 414 | 416 | 337.07 | 362.20 |
| 95 | mmu-miR-210-3p | 413 | 451 | 336.26 | 392.68 |
| 96 | mmu-miR-133a-3p | 408 | 335 | 332.19 | 291.68 |
| 97 | mmu-miR-21a-3p | 404 | 303 | 328.93 | 263.82 |
| 98 | mmu-miR-139-5p | 395 | 344 | 321.60 | 299.51 |
| 99 | mmu-miR-151-5p | 389 | 381 | 316.72 | 331.73 |
| 100 | mmu-miR-652-3p | 351 | 397 | 285.78 | 345.66 |
| 101 | mmu-miR-15b-3p | 341 | 300 | 277.64 | 261.20 |
| 102 | mmu-miR-421-3p | 323 | 306 | 262.98 | 266.43 |
| 103 | mmu-miR-29c-3p | 323 | 177 | 262.98 | 154.11 |
| 104 | mmu-miR-342-3p | 311 | 255 | 253.21 | 222.02 |
| 105 | mmu-miR-101b-3p | 309 | 278 | 251.58 | 242.05 |
| 106 | mmu-miR-205-5p | 308 | 413 | 250.77 | 359.59 |
| 107 | mmu-miR-429-3p | 302 | 227 | 245.88 | 197.64 |
| 108 | mmu-miR-200a-3p | 283 | 174 | 230.41 | 151.50 |
| 109 | mmu-miR-17-5p | 277 | 214 | 225.53 | 186.33 |
| 110 | mmu-miR-26b-5p | 275 | 261 | 223.90 | 227.25 |
| 111 | mmu-miR-148b-3p | 275 | 217 | 223.90 | 188.94 |
| 112 | mmu-miR-6240 | 271 | 267 | 220.64 | 232.47 |
| 113 | mmu-miR-30a-3p | 267 | 222 | 217.39 | 193.29 |
| 114 | mmu-miR-100-5p | 266 | 263 | 216.57 | 228.99 |
| 115 | mmu-miR-181d-5p | 238 | 232 | 193.78 | 202.00 |
| 116 | mmu-miR-298-5p | 236 | 281 | 192.15 | 244.66 |
| 117 | mmu-miR-3473e | 236 | 230 | 192.15 | 200.26 |
| 118 | mmu-miR-410-3p | 235 | 118 | 191.33 | 102.74 |
| 119 | mmu-miR-3473b | 232 | 229 | 188.89 | 199.39 |
| 120 | mmu-miR-199a-5p | 228 | 277 | 185.63 | 241.18 |
| 121 | mmu-miR-300-3p | 215 | 104 | 175.05 | 90.55 |
| 122 | mmu-miR-136-5p | 206 | 234 | 167.72 | 203.74 |
| 123 | mmu-miR-194-5p | 197 | 100 | 160.39 | 87.07 |
| 124 | mmu-miR-351-5p | 185 | 253 | 150.62 | 220.28 |
| 125 | mmu-miR-99a-5p | 184 | 182 | 149.81 | 158.46 |
| 126 | mmu-miR-145a-3p | 179 | 177 | 145.74 | 154.11 |
| 127 | mmu-miR-361-5p | 177 | 160 | 144.11 | 139.31 |
| 128 | mmu-miR-674-3p | 168 | 178 | 136.78 | 154.98 |
| 129 | mmu-miR-872-5p | 168 | 159 | 136.78 | 138.44 |
| 130 | mmu-miR-16-2-3p | 166 | 137 | 135.16 | 119.28 |
| 131 | mmu-miR-122-5p | 164 | 27 | 133.53 | 23.51 |
| 132 | mmu-miR-98-5p | 162 | 133 | 131.90 | 115.80 |
| 133 | mmu-miR-138-5p | 156 | 53 | 127.01 | 46.15 |
| 134 | mmu-miR-19a-3p | 149 | 133 | 121.31 | 115.80 |
| 135 | mmu-miR-148a-5p | 141 | 113 | 114.80 | 98.39 |
| 136 | mmu-miR-32-5p | 135 | 127 | 109.92 | 110.58 |
| 137 | mmu-miR-125b-2-3p | 134 | 146 | 109.10 | 127.12 |
| 138 | mmu-miR-20a-5p | 133 | 140 | 108.29 | 121.90 |
| 139 | mmu-miR-5126 | 131 | 112 | 106.66 | 97.52 |
| 140 | mmu-miR-24-3p | 128 | 137 | 104.22 | 119.28 |
| 141 | mmu-miR-541-5p | 125 | 113 | 101.77 | 98.39 |
| 142 | mmu-miR-381-3p | 120 | 124 | 97.70 | 107.96 |
| 143 | mmu-miR-148b-5p | 117 | 103 | 95.26 | 89.68 |
| 144 | mmu-miR-145a-5p | 114 | 100 | 92.82 | 87.07 |
| 145 | mmu-miR-872-3p | 113 | 101 | 92.00 | 87.94 |
| 146 | mmu-miR-183-5p | 110 | 30 | 89.56 | 26.12 |
| 147 | mmu-miR-132-3p | 108 | 52 | 87.93 | 45.28 |
| 148 | mmu-miR-363-3p | 105 | 167 | 85.49 | 145.40 |
| 149 | mmu-miR-322-3p | 105 | 140 | 85.49 | 121.90 |
| 150 | mmu-miR-22-5p | 99 | 81 | 80.60 | 70.53 |
| 151 | mmu-miR-322-5p | 98 | 104 | 79.79 | 90.55 |
| 152 | mmu-miR-335-5p | 90 | 87 | 73.28 | 75.75 |
| 153 | mmu-miR-339-5p | 90 | 83 | 73.28 | 72.27 |
| 154 | mmu-miR-1843a-5p | 88 | 74 | 71.65 | 64.43 |
| 155 | mmu-let-7e-5p | 86 | 71 | 70.02 | 61.82 |
| 156 | mmu-miR-9-5p | 85 | 26 | 69.21 | 22.64 |
| 157 | mmu-miR-29b-3p | 84 | 43 | 68.39 | 37.44 |
| 158 | mmu-miR-142a-3p | 83 | 69 | 67.58 | 60.08 |
| 159 | mmu-miR-455-5p | 81 | 117 | 65.95 | 101.87 |
| 160 | mmu-miR-361-3p | 79 | 83 | 64.32 | 72.27 |
| 161 | mmu-miR-214-5p | 78 | 95 | 63.51 | 82.71 |
| 162 | mmu-miR-501-3p | 77 | 53 | 62.69 | 46.15 |
| 163 | mmu-miR-1964-3p | 74 | 75 | 60.25 | 65.30 |
| 164 | mmu-miR-499-5p | 71 | 47 | 57.81 | 40.92 |
| 165 | mmu-miR-214-3p | 69 | 97 | 56.18 | 84.46 |
| 166 | mmu-miR-17-3p | 66 | 69 | 53.74 | 60.08 |
| 167 | mmu-miR-1843b-5p | 62 | 42 | 50.48 | 36.57 |
| 168 | mmu-miR-208a-3p | 62 | 37 | 50.48 | 32.22 |
| 169 | mmu-miR-215-3p | 61 | 60 | 49.67 | 52.24 |
| 170 | mmu-miR-8114 | 60 | 65 | 48.85 | 56.59 |
| 171 | mmu-miR-181a-1-3p | 59 | 71 | 48.04 | 61.82 |
| 172 | mmu-miR-218-5p | 59 | 44 | 48.04 | 38.31 |
| 173 | mmu-miR-193b-3p | 58 | 79 | 47.22 | 68.78 |
| 174 | mmu-miR-133b-3p | 58 | 40 | 47.22 | 34.83 |
| 175 | mmu-miR-450b-5p | 57 | 50 | 46.41 | 43.53 |
| 176 | mmu-miR-365-3p | 56 | 61 | 45.59 | 53.11 |
| 177 | mmu-miR-676-5p | 53 | 56 | 43.15 | 48.76 |
| 178 | mmu-miR-664-3p | 53 | 54 | 43.15 | 47.02 |
| 179 | mmu-miR-18a-5p | 53 | 48 | 43.15 | 41.79 |
| 180 | mmu-miR-5121 | 53 | 27 | 43.15 | 23.51 |
| 181 | mmu-miR-28a-3p | 52 | 45 | 42.34 | 39.18 |
| 182 | mmu-miR-668-3p | 52 | 3 | 42.34 | 2.61 |
| 183 | mmu-miR-222-3p | 50 | 50 | 40.71 | 43.53 |
| 184 | mmu-miR-802-3p | 48 | 5 | 39.08 | 4.35 |
| 185 | mmu-miR-411-5p | 47 | 53 | 38.27 | 46.15 |
| 186 | mmu-miR-434-5p | 46 | 50 | 37.45 | 43.53 |
| 187 | mmu-miR-208b-3p | 44 | 44 | 35.82 | 38.31 |
| 188 | mmu-miR-30e-3p | 44 | 44 | 35.82 | 38.31 |
| 189 | mmu-miR-378a-5p | 41 | 42 | 33.38 | 36.57 |
| 190 | mmu-miR-1981-5p | 41 | 37 | 33.38 | 32.22 |
| 191 | mmu-miR-30d-3p | 41 | 37 | 33.38 | 32.22 |
| 192 | mmu-miR-339-3p | 41 | 31 | 33.38 | 26.99 |
| 193 | mmu-miR-125b-1-3p | 40 | 39 | 32.57 | 33.96 |
| 194 | mmu-miR-10a-3p | 40 | 36 | 32.57 | 31.34 |
| 195 | mmu-miR-326-3p | 40 | 31 | 32.57 | 26.99 |
| 196 | mmu-miR-409-3p | 39 | 46 | 31.75 | 40.05 |
| 197 | mmu-miR-136-3p | 39 | 34 | 31.75 | 29.60 |
| 198 | mmu-miR-28a-5p | 39 | 34 | 31.75 | 29.60 |
| 199 | mmu-let-7i-3p | 38 | 47 | 30.94 | 40.92 |
| 200 | mmu-miR-450a-5p | 38 | 47 | 30.94 | 40.92 |
| 201 | mmu-let-7a-1-3p>mmu-let-7c-2-3p | 38 | 29 | 30.94 | 25.25 |
| 202 | mmu-miR-212-3p | 38 | 19 | 30.94 | 16.54 |
| 203 | mmu-miR-129b-5p | 38 | 2 | 30.94 | 1.74 |
| 204 | mmu-miR-30f | 37 | 46 | 30.12 | 40.05 |
| 205 | mmu-miR-669a-5p>mmu-miR-669p-5p | 36 | 40 | 29.31 | 34.83 |
| 206 | mmu-miR-181c-3p | 36 | 31 | 29.31 | 26.99 |
| 207 | mmu-miR-34a-5p | 36 | 22 | 29.31 | 19.16 |
| 208 | mmu-miR-15a-3p | 35 | 30 | 28.50 | 26.12 |
| 209 | mmu-miR-187-3p | 34 | 14 | 27.68 | 12.19 |
| 210 | mmu-miR-130b-5p | 33 | 33 | 26.87 | 28.73 |
| 211 | mmu-miR-671-3p | 33 | 24 | 26.87 | 20.90 |
| 212 | mmu-miR-129-2-3p | 33 | 2 | 26.87 | 1.74 |
| 213 | mmu-miR-152-5p | 32 | 38 | 26.05 | 33.09 |
| 214 | mmu-miR-140-5p | 31 | 46 | 25.24 | 40.05 |
| 215 | mmu-miR-296-5p | 31 | 26 | 25.24 | 22.64 |
| 216 | mmu-miR-511-3p | 29 | 15 | 23.61 | 13.06 |
| 217 | mmu-miR-199b-5p | 28 | 47 | 22.80 | 40.92 |
| 218 | mmu-miR-329-5p | 28 | 37 | 22.80 | 32.22 |
| 219 | mmu-miR-3068-3p | 28 | 31 | 22.80 | 26.99 |
| 220 | mmu-miR-33-5p | 28 | 30 | 22.80 | 26.12 |
| 221 | mmu-miR-223-5p | 28 | 25 | 22.80 | 21.77 |
| 222 | mmu-miR-433-3p | 28 | 10 | 22.80 | 8.71 |
| 223 | mmu-miR-18a-3p | 27 | 25 | 21.98 | 21.77 |
| 224 | mmu-miR-3057-5p | 27 | 19 | 21.98 | 16.54 |
| 225 | mmu-miR-6538 | 26 | 30 | 21.17 | 26.12 |
| 226 | mmu-miR-195a-5p | 26 | 23 | 21.17 | 20.03 |
| 227 | mmu-miR-98-3p | 26 | 20 | 21.17 | 17.41 |
| 228 | mmu-miR-34c-5p | 25 | 25 | 20.35 | 21.77 |
| 229 | mmu-miR-129b-3p | 25 | 4 | 20.35 | 3.48 |
| 230 | mmu-miR-219b-3p | 24 | 13 | 19.54 | 11.32 |
| 231 | mmu-miR-210-5p | 23 | 26 | 18.73 | 22.64 |
| 232 | mmu-miR-1306-5p | 23 | 23 | 18.73 | 20.03 |
| 233 | mmu-miR-615-3p | 23 | 22 | 18.73 | 19.16 |
| 234 | mmu-miR-204-5p | 22 | 25 | 17.91 | 21.77 |
| 235 | mmu-miR-431-5p | 22 | 24 | 17.91 | 20.90 |
| 236 | mmu-miR-3071-5p | 22 | 19 | 17.91 | 16.54 |
| 237 | mmu-miR-193a-3p | 22 | 15 | 17.91 | 13.06 |
| 238 | mmu-miR-31-5p | 21 | 27 | 17.10 | 23.51 |
| 239 | mmu-miR-338-3p | 21 | 21 | 17.10 | 18.28 |
| 240 | mmu-miR-191-3p | 21 | 20 | 17.10 | 17.41 |
| 241 | mmu-miR-1843a-3p | 21 | 16 | 17.10 | 13.93 |
| 242 | mmu-miR-362-3p | 20 | 26 | 16.28 | 22.64 |
| 243 | mmu-miR-574-5p | 20 | 23 | 16.28 | 20.03 |
| 244 | mmu-miR-301b-3p | 20 | 22 | 16.28 | 19.16 |
| 245 | mmu-miR-708-3p | 20 | 15 | 16.28 | 13.06 |
| 246 | mmu-miR-374b-5p | 20 | 13 | 16.28 | 11.32 |
| 247 | mmu-miR-674-5p | 20 | 13 | 16.28 | 11.32 |
| 248 | mmu-miR-7a-1-3p | 19 | 19 | 15.47 | 16.54 |
| 249 | mmu-miR-1249-3p | 18 | 13 | 14.66 | 11.32 |
| 250 | mmu-miR-153-3p | 18 | 1 | 14.66 | 0.87 |
| 251 | mmu-miR-350-3p | 17 | 17 | 13.84 | 14.80 |
| 252 | mmu-miR-222-5p | 17 | 14 | 13.84 | 12.19 |
| 253 | mmu-miR-26b-3p | 17 | 12 | 13.84 | 10.45 |
| 254 | mmu-miR-6395 | 17 | 11 | 13.84 | 9.58 |
| 255 | mmu-let-7b-3p | 17 | 11 | 13.84 | 9.58 |
| 256 | mmu-miR-369-3p | 17 | 5 | 13.84 | 4.35 |
| 257 | mmu-miR-542-5p | 16 | 24 | 13.03 | 20.90 |
| 258 | mmu-miR-99b-3p | 16 | 19 | 13.03 | 16.54 |
| 259 | mmu-miR-296-3p | 16 | 17 | 13.03 | 14.80 |
| 260 | mmu-miR-532-3p | 16 | 16 | 13.03 | 13.93 |
| 261 | mmu-miR-101a-5p | 16 | 10 | 13.03 | 8.71 |
| 262 | mmu-miR-147-3p | 16 | 10 | 13.03 | 8.71 |
| 263 | mmu-miR-185-5p | 16 | 8 | 13.03 | 6.97 |
| 264 | mmu-miR-134-5p | 15 | 18 | 12.21 | 15.67 |
| 265 | mmu-miR-1191a | 15 | 15 | 12.21 | 13.06 |
| 266 | mmu-miR-335-3p | 15 | 14 | 12.21 | 12.19 |
| 267 | mmu-miR-802-5p | 15 | 9 | 12.21 | 7.84 |
| 268 | mmu-miR-1247-5p | 15 | 9 | 12.21 | 7.84 |
| 269 | mmu-miR-150-3p | 14 | 15 | 11.40 | 13.06 |
| 270 | mmu-miR-338-5p | 14 | 13 | 11.40 | 11.32 |
| 271 | mmu-miR-1839-3p | 14 | 11 | 11.40 | 9.58 |
| 272 | mmu-miR-744-3p | 14 | 11 | 11.40 | 9.58 |
| 273 | mmu-miR-344d-3p | 14 | 1 | 11.40 | 0.87 |
| 274 | mmu-miR-30c-2-3p | 13 | 18 | 10.58 | 15.67 |
| 275 | mmu-miR-25-5p | 13 | 16 | 10.58 | 13.93 |
| 276 | mmu-miR-592-5p | 13 | 15 | 10.58 | 13.06 |
| 277 | mmu-miR-673-5p | 13 | 12 | 10.58 | 10.45 |
| 278 | mmu-miR-342-5p | 13 | 12 | 10.58 | 10.45 |
| 279 | mmu-miR-126b-5p | 13 | 11 | 10.58 | 9.58 |
| 280 | mmu-miR-382-5p | 13 | 6 | 10.58 | 5.22 |
| 281 | mmu-miR-5099 | 13 | 5 | 10.58 | 4.35 |
| 282 | mmu-miR-330-3p | 13 | 3 | 10.58 | 2.61 |
| 283 | mmu-miR-127-5p | 13 | 2 | 10.58 | 1.74 |
| 284 | mmu-miR-1927 | 12 | 14 | 9.77 | 12.19 |
| 285 | mmu-miR-324-5p | 12 | 11 | 9.77 | 9.58 |
| 286 | mmu-miR-200b-3p | 12 | 8 | 9.77 | 6.97 |
| 287 | mmu-miR-330-5p | 12 | 8 | 9.77 | 6.97 |
| 288 | mmu-miR-3473d | 12 | 6 | 9.77 | 5.22 |
| 289 | mmu-miR-341-3p | 12 | 1 | 9.77 | 0.87 |
| 290 | mmu-miR-16-1-3p | 11 | 16 | 8.96 | 13.93 |
| 291 | mmu-miR-218-1-3p | 11 | 12 | 8.96 | 10.45 |
| 292 | mmu-miR-582-3p | 11 | 10 | 8.96 | 8.71 |
| 293 | mmu-miR-1195 | 11 | 9 | 8.96 | 7.84 |
| 294 | mmu-miR-380-3p | 11 | 7 | 8.96 | 6.09 |
| 295 | mmu-miR-203-5p | 11 | 6 | 8.96 | 5.22 |
| 296 | mmu-miR-195a-3p | 10 | 16 | 8.14 | 13.93 |
| 297 | mmu-miR-542-3p | 10 | 14 | 8.14 | 12.19 |
| 298 | mmu-miR-3071-3p | 10 | 9 | 8.14 | 7.84 |
| 299 | mmu-miR-125a-3p | 10 | 8 | 8.14 | 6.97 |
| 300 | mmu-miR-669a-3p>mmu-miR-669o-3p | 9 | 12 | 7.33 | 10.45 |
| 301 | mmu-miR-1306-3p | 9 | 10 | 7.33 | 8.71 |
| 302 | mmu-miR-503-3p | 9 | 10 | 7.33 | 8.71 |
| 303 | mmu-miR-99a-3p | 9 | 9 | 7.33 | 7.84 |
| 304 | mmu-miR-146b-5p | 9 | 9 | 7.33 | 7.84 |
| 305 | mmu-miR-19a-5p | 9 | 9 | 7.33 | 7.84 |
| 306 | mmu-miR-1983 | 9 | 7 | 7.33 | 6.09 |
| 307 | mmu-miR-598-3p | 9 | 6 | 7.33 | 5.22 |
| 308 | mmu-miR-1943-5p | 9 | 5 | 7.33 | 4.35 |
| 309 | mmu-miR-879-5p | 9 | 2 | 7.33 | 1.74 |
| 310 | mmu-miR-96-5p | 8 | 10 | 6.51 | 8.71 |
| 311 | mmu-miR-200c-3p | 8 | 10 | 6.51 | 8.71 |
| 312 | mmu-miR-3058-3p | 8 | 9 | 6.51 | 7.84 |
| 313 | mmu-miR-24-1-5p | 8 | 8 | 6.51 | 6.97 |
| 314 | mmu-miR-93-3p | 8 | 6 | 6.51 | 5.22 |
| 315 | mmu-let-7e-3p | 8 | 3 | 6.51 | 2.61 |
| 316 | mmu-miR-196b-5p | 8 | 3 | 6.51 | 2.61 |
| 317 | mmu-miR-877-3p | 7 | 8 | 5.70 | 6.97 |
| 318 | mmu-miR-3065-3p | 7 | 8 | 5.70 | 6.97 |
| 319 | mmu-miR-331-5p | 7 | 8 | 5.70 | 6.97 |
| 320 | mmu-miR-20b-5p | 7 | 7 | 5.70 | 6.09 |
| 321 | mmu-miR-376b-3p | 7 | 7 | 5.70 | 6.09 |
| 322 | mmu-miR-700-5p | 7 | 5 | 5.70 | 4.35 |
| 323 | mmu-miR-6944-3p | 7 | 4 | 5.70 | 3.48 |
| 324 | mmu-miR-1947-5p | 7 | 3 | 5.70 | 2.61 |
| 325 | mmu-miR-154-5p | 7 | 3 | 5.70 | 2.61 |
| 326 | mmu-miR-487b-3p | 7 | 3 | 5.70 | 2.61 |
| 327 | mmu-miR-714 | 7 | 3 | 5.70 | 2.61 |
| 328 | mmu-miR-128-1-5p | 7 | 1 | 5.70 | 0.87 |
| 329 | mmu-miR-1191b-5p | 6 | 10 | 4.89 | 8.71 |
| 330 | mmu-miR-205-3p | 6 | 10 | 4.89 | 8.71 |
| 331 | mmu-miR-1960 | 6 | 8 | 4.89 | 6.97 |
| 332 | mmu-miR-216a-5p | 6 | 7 | 4.89 | 6.09 |
| 333 | mmu-let-7f-1-3p | 6 | 6 | 4.89 | 5.22 |
| 334 | mmu-miR-3112-5p | 6 | 5 | 4.89 | 4.35 |
| 335 | mmu-miR-500-3p | 6 | 4 | 4.89 | 3.48 |
| 336 | mmu-miR-3105-3p | 6 | 4 | 4.89 | 3.48 |
| 337 | mmu-miR-340-3p | 6 | 3 | 4.89 | 2.61 |
| 338 | mmu-miR-467d-5p | 6 | 3 | 4.89 | 2.61 |
| 339 | mmu-miR-323-3p | 6 | 3 | 4.89 | 2.61 |
| 340 | mmu-miR-582-5p | 6 | 3 | 4.89 | 2.61 |
| 341 | mmu-miR-92a-1-5p | 6 | 3 | 4.89 | 2.61 |
| 342 | mmu-miR-425-3p | 6 | 2 | 4.89 | 1.74 |
| 343 | mmu-miR-129-1-3p | 6 | 1 | 4.89 | 0.87 |
| 344 | mmu-miR-672-5p | 5 | 8 | 4.07 | 6.97 |
| 345 | mmu-miR-1930-5p | 5 | 7 | 4.07 | 6.09 |
| 346 | mmu-miR-196b-3p | 5 | 6 | 4.07 | 5.22 |
| 347 | mmu-miR-376c-3p | 5 | 6 | 4.07 | 5.22 |
| 348 | mmu-miR-7658-3p | 5 | 6 | 4.07 | 5.22 |
| 349 | mmu-miR-690 | 5 | 6 | 4.07 | 5.22 |
| 350 | mmu-miR-877-5p | 5 | 6 | 4.07 | 5.22 |
| 351 | mmu-miR-106a-5p | 5 | 5 | 4.07 | 4.35 |
| 352 | mmu-miR-505-3p | 5 | 5 | 4.07 | 4.35 |
| 353 | mmu-miR-5119 | 5 | 5 | 4.07 | 4.35 |
| 354 | mmu-miR-708-5p | 5 | 5 | 4.07 | 4.35 |
| 355 | mmu-miR-1934-5p | 5 | 4 | 4.07 | 3.48 |
| 356 | mmu-miR-378b | 5 | 4 | 4.07 | 3.48 |
| 357 | mmu-miR-155-5p | 5 | 4 | 4.07 | 3.48 |
| 358 | mmu-miR-3963 | 5 | 4 | 4.07 | 3.48 |
| 359 | mmu-miR-7687-5p | 5 | 4 | 4.07 | 3.48 |
| 360 | mmu-miR-1933-3p | 5 | 3 | 4.07 | 2.61 |
| 361 | mmu-miR-1968-5p | 5 | 3 | 4.07 | 2.61 |
| 362 | mmu-miR-183-3p | 5 | 2 | 4.07 | 1.74 |
| 363 | mmu-miR-219a-2-3p | 5 | 1 | 4.07 | 0.87 |
| 364 | mmu-miR-666-3p | 5 | 1 | 4.07 | 0.87 |
| 365 | mmu-miR-449a-5p | 4 | 7 | 3.26 | 6.09 |
| 366 | mmu-miR-224-5p | 4 | 6 | 3.26 | 5.22 |
| 367 | mmu-miR-27b-5p | 4 | 6 | 3.26 | 5.22 |
| 368 | mmu-miR-297a-5p | 4 | 6 | 3.26 | 5.22 |
| 369 | mmu-miR-6911-3p | 4 | 6 | 3.26 | 5.22 |
| 370 | mmu-miR-485-5p | 4 | 5 | 3.26 | 4.35 |
| 371 | mmu-miR-10b-3p | 4 | 5 | 3.26 | 4.35 |
| 372 | mmu-miR-3074-1-3p | 4 | 4 | 3.26 | 3.48 |
| 373 | mmu-miR-665-3p | 4 | 4 | 3.26 | 3.48 |
| 374 | mmu-miR-9769-3p | 4 | 4 | 3.26 | 3.48 |
| 375 | mmu-miR-3074-2-3p | 4 | 3 | 3.26 | 2.61 |
| 376 | mmu-miR-3102-3p.2-3p | 4 | 3 | 3.26 | 2.61 |
| 377 | mmu-miR-874-3p | 4 | 3 | 3.26 | 2.61 |
| 378 | mmu-miR-337-5p | 4 | 2 | 3.26 | 1.74 |
| 379 | mmu-miR-1948-3p | 4 | 1 | 3.26 | 0.87 |
| 380 | mmu-miR-3470b | 4 | 1 | 3.26 | 0.87 |
| 381 | mmu-miR-369-5p | 4 | 1 | 3.26 | 0.87 |
| 382 | mmu-miR-370-3p | 4 | 1 | 3.26 | 0.87 |
| 383 | mmu-miR-29a-5p | 4 | 1 | 3.26 | 0.87 |
| 384 | mmu-miR-7063-5p | 4 | 1 | 3.26 | 0.87 |
| 385 | mmu-miR-26a-2-3p | 3 | 5 | 2.44 | 4.35 |
| 386 | mmu-miR-3535 | 3 | 5 | 2.44 | 4.35 |
| 387 | mmu-miR-669d-5p | 3 | 5 | 2.44 | 4.35 |
| 388 | mmu-miR-3102-3p | 3 | 5 | 2.44 | 4.35 |
| 389 | mmu-miR-503-5p | 3 | 5 | 2.44 | 4.35 |
| 390 | mmu-miR-6955-3p | 3 | 5 | 2.44 | 4.35 |
| 391 | mmu-miR-3082-3p | 3 | 4 | 2.44 | 3.48 |
| 392 | mmu-miR-376b-5p | 3 | 4 | 2.44 | 3.48 |
| 393 | mmu-miR-184-3p | 3 | 4 | 2.44 | 3.48 |
| 394 | mmu-miR-196a-5p | 3 | 4 | 2.44 | 3.48 |
| 395 | mmu-miR-8097 | 3 | 4 | 2.44 | 3.48 |
| 396 | mmu-let-7j | 3 | 3 | 2.44 | 2.61 |
| 397 | mmu-miR-3084-3p | 3 | 3 | 2.44 | 2.61 |
| 398 | mmu-miR-6236 | 3 | 3 | 2.44 | 2.61 |
| 399 | mmu-miR-7661-3p | 3 | 3 | 2.44 | 2.61 |
| 400 | mmu-miR-193b-5p | 3 | 3 | 2.44 | 2.61 |
| 401 | mmu-miR-324-3p | 3 | 3 | 2.44 | 2.61 |
| 402 | mmu-miR-6948-3p | 3 | 3 | 2.44 | 2.61 |
| 403 | mmu-miR-6988-3p | 3 | 3 | 2.44 | 2.61 |
| 404 | mmu-miR-3103-3p | 3 | 2 | 2.44 | 1.74 |
| 405 | mmu-miR-6960-5p | 3 | 2 | 2.44 | 1.74 |
| 406 | mmu-miR-871-3p | 3 | 2 | 2.44 | 1.74 |
| 407 | mmu-let-7c-1-3p | 3 | 1 | 2.44 | 0.87 |
| 408 | mmu-miR-137-3p | 3 | 1 | 2.44 | 0.87 |
| 409 | mmu-miR-200a-5p | 3 | 1 | 2.44 | 0.87 |
| 410 | mmu-miR-219a-1-3p | 3 | 1 | 2.44 | 0.87 |
| 411 | mmu-miR-3065-5p | 3 | 1 | 2.44 | 0.87 |
| 412 | mmu-miR-7015-3p | 3 | 1 | 2.44 | 0.87 |
| 413 | mmu-miR-8103 | 3 | 1 | 2.44 | 0.87 |
| 414 | mmu-miR-299a-3p | 3 | 1 | 2.44 | 0.87 |
| 415 | mmu-miR-485-3p | 3 | 1 | 2.44 | 0.87 |
| 416 | mmu-miR-5100 | 3 | 1 | 2.44 | 0.87 |
| 417 | mmu-miR-702-3p | 3 | 1 | 2.44 | 0.87 |
| 418 | mmu-miR-709 | 3 | 1 | 2.44 | 0.87 |
| 419 | mmu-miR-7235-3p | 3 | 1 | 2.44 | 0.87 |
| 420 | mmu-miR-466d-5p | 2 | 3 | 1.63 | 2.61 |
| 421 | mmu-miR-8105 | 2 | 3 | 1.63 | 2.61 |
| 422 | mmu-miR-299a-5p | 2 | 3 | 1.63 | 2.61 |
| 423 | mmu-miR-3473g | 2 | 3 | 1.63 | 2.61 |
| 424 | mmu-miR-465c-5p | 2 | 3 | 1.63 | 2.61 |
| 425 | mmu-miR-6418-3p | 2 | 3 | 1.63 | 2.61 |
| 426 | mmu-miR-6938-3p | 2 | 3 | 1.63 | 2.61 |
| 427 | mmu-miR-6967-3p | 2 | 3 | 1.63 | 2.61 |
| 428 | mmu-miR-7035-3p | 2 | 3 | 1.63 | 2.61 |
| 429 | mmu-miR-18b-3p | 2 | 2 | 1.63 | 1.74 |
| 430 | mmu-miR-337-3p | 2 | 2 | 1.63 | 1.74 |
| 431 | mmu-miR-490-3p | 2 | 2 | 1.63 | 1.74 |
| 432 | mmu-miR-7653-5p | 2 | 2 | 1.63 | 1.74 |
| 433 | mmu-miR-130a-5p | 2 | 2 | 1.63 | 1.74 |
| 434 | mmu-miR-3470a | 2 | 2 | 1.63 | 1.74 |
| 435 | mmu-miR-466g | 2 | 2 | 1.63 | 1.74 |
| 436 | mmu-let-7f-2-3p | 2 | 1 | 1.63 | 0.87 |
| 437 | mmu-miR-1954 | 2 | 1 | 1.63 | 0.87 |
| 438 | mmu-miR-3061-3p | 2 | 1 | 1.63 | 0.87 |
| 439 | mmu-miR-34b-3p | 2 | 1 | 1.63 | 0.87 |
| 440 | mmu-miR-376a-5p | 2 | 1 | 1.63 | 0.87 |
| 441 | mmu-miR-378d | 2 | 1 | 1.63 | 0.87 |
| 442 | mmu-miR-1199-5p | 2 | 1 | 1.63 | 0.87 |
| 443 | mmu-miR-146b-3p | 2 | 1 | 1.63 | 0.87 |
| 444 | mmu-miR-30c-1-3p | 2 | 1 | 1.63 | 0.87 |
| 445 | mmu-miR-345-5p | 2 | 1 | 1.63 | 0.87 |
| 446 | mmu-miR-466d-3p | 2 | 1 | 1.63 | 0.87 |
| 447 | mmu-miR-5128 | 2 | 1 | 1.63 | 0.87 |
| 448 | mmu-miR-693-3p | 2 | 1 | 1.63 | 0.87 |
| 449 | mmu-miR-132-5p | 1 | 2 | 0.81 | 1.74 |
| 450 | mmu-miR-139-3p | 1 | 2 | 0.81 | 1.74 |
| 451 | mmu-miR-194-2-3p | 1 | 2 | 0.81 | 1.74 |
| 452 | mmu-miR-1950 | 1 | 2 | 0.81 | 1.74 |
| 453 | mmu-miR-1970 | 1 | 2 | 0.81 | 1.74 |
| 454 | mmu-miR-201-5p | 1 | 2 | 0.81 | 1.74 |
| 455 | mmu-miR-20b-3p | 1 | 2 | 0.81 | 1.74 |
| 456 | mmu-miR-3088-3p | 1 | 2 | 0.81 | 1.74 |
| 457 | mmu-miR-3091-5p | 1 | 2 | 0.81 | 1.74 |
| 458 | mmu-miR-3098-5p | 1 | 2 | 0.81 | 1.74 |
| 459 | mmu-miR-32-3p | 1 | 2 | 0.81 | 1.74 |
| 460 | mmu-miR-3544-3p | 1 | 2 | 0.81 | 1.74 |
| 461 | mmu-miR-363-5p | 1 | 2 | 0.81 | 1.74 |
| 462 | mmu-miR-467a-5p | 1 | 2 | 0.81 | 1.74 |
| 463 | mmu-miR-467c-5p | 1 | 2 | 0.81 | 1.74 |
| 464 | mmu-miR-6541 | 1 | 2 | 0.81 | 1.74 |
| 465 | mmu-miR-671-5p | 1 | 2 | 0.81 | 1.74 |
| 466 | mmu-miR-6947-3p | 1 | 2 | 0.81 | 1.74 |
