# Supplementary material for: Characterization of mouse serum exosomal small RNA content: The origins and their roles in modulating inflammatory response
Source: Oncotarget. 2017 Apr 27;8(26):42712–27. doi: 10.18632/oncotarget.17448 (PMC5522100; doi:10.18632/oncotarget.17448)
Supplement: Supplementary file 3 [file oncotarget-08-42712-s003.docx]

|  | Symbol | Entrez Gene Name | Location | Type(s) |
| --- | --- | --- | --- | --- |
| 1 | AFF3 | AF4/FMR2 family member 3 | Nucleus | transcription regulator |
| 2 | AKTIP | AKT interacting protein | Cytoplasm | other |
| 3 | ANLN | anillin actin binding protein | Cytoplasm | other |
| 4 | APP | amyloid beta precursor protein | Plasma Membrane | other |
| 5 | ARID4B | AT-rich interaction domain 4B | Nucleus | enzyme |
| 6 | ATF6 | activating transcription factor 6 | Cytoplasm | transcription regulator |
| 7 | BAMBI | BMP and activin membrane bound inhibitor | Plasma Membrane | other |
| 8 | BAX | BCL2 associated X, apoptosis regulator | Cytoplasm | transporter |
| 9 | BBC3 | BCL2 binding component 3 | Cytoplasm | other |
| 10 | BCL2 | BCL2, apoptosis regulator | Cytoplasm | transporter |
| 11 | BCL2L1 | BCL2 like 1 | Cytoplasm | other |
| 12 | BCL2L2 | BCL2 like 2 | Cytoplasm | other |
| 13 | BCL6 | B-cell CLL/lymphoma 6 | Nucleus | transcription regulator |
| 14 | BNIP2 | BCL2 interacting protein 2 | Cytoplasm | other |
| 15 | BSG | basigin (Ok blood group) | Plasma Membrane | transporter |
| 16 | CCKBR | cholecystokinin B receptor | Plasma Membrane | G-protein coupled receptor |
| 17 | Ccl9 | chemokine (C-C motif) ligand 9 | Extracellular Space | cytokine |
| 18 | CCND1 | cyclin D1 | Nucleus | transcription regulator |
| 19 | CCNE1 | cyclin E1 | Nucleus | transcription regulator |
| 20 | CCNE2 | cyclin E2 | Nucleus | other |
| 21 | CCNF | cyclin F | Nucleus | other |
| 22 | CDC14A | cell division cycle 14A | Nucleus | phosphatase |
| 23 | CDC25A | cell division cycle 25A | Nucleus | phosphatase |
| 24 | CDKN1A | cyclin dependent kinase inhibitor 1A | Nucleus | kinase |
| 25 | CHEK1 | checkpoint kinase 1 | Nucleus | kinase |
| 26 | COL1A2 | collagen type I alpha 2 chain | Extracellular Space | other |
| 27 | CREB1 | cAMP responsive element binding protein 1 | Nucleus | transcription regulator |
| 28 | CRIM1 | cysteine rich transmembrane BMP regulator 1 | Extracellular Space | kinase |
| 29 | CRP | C-reactive protein, pentraxin-related | Extracellular Space | other |
| 30 | CTGF | connective tissue growth factor | Extracellular Space | growth factor |
| 31 | E2F1 | E2F transcription factor 1 | Nucleus | transcription regulator |
| 32 | E2F2 | E2F transcription factor 2 | Nucleus | transcription regulator |
| 33 | E2F3 | E2F transcription factor 3 | Nucleus | transcription regulator |
| 34 | FASLG | Fas ligand | Extracellular Space | cytokine |
| 35 | FBXO33 | F-box protein 33 | Other | other |
| 36 | FBXW7 | F-box and WD repeat domain containing 7 | Nucleus | transcription regulator |
| 37 | FBXW7 | F-box and WD repeat domain containing 7 | Nucleus | transcription regulator |
| 38 | FGF2 | fibroblast growth factor 2 | Extracellular Space | growth factor |
| 39 | FGF7 | fibroblast growth factor 7 | Extracellular Space | growth factor |
| 40 | FGFR1 | fibroblast growth factor receptor 1 | Plasma Membrane | kinase |
| 41 | FLT3 | fms related tyrosine kinase 3 | Plasma Membrane | kinase |
| 42 | FOXO1 | forkhead box O1 | Nucleus | transcription regulator |
| 43 | GRB10 | growth factor receptor bound protein 10 | Cytoplasm | other |
| 44 | GRB2 | growth factor receptor bound protein 2 | Cytoplasm | kinase |
| 45 | HBP1 | HMG-box transcription factor 1 | Nucleus | transcription regulator |
| 46 | HMGA2 | high mobility group AT-hook 2 | Nucleus | enzyme |
| 47 | Hmga2 | high mobility group AT-hook 2 | Nucleus | enzyme |
| 48 | IFI16 | interferon gamma inducible protein 16 | Nucleus | transcription regulator |
| 49 | IGF1 | insulin like growth factor 1 | Extracellular Space | growth factor |
| 50 | IGF1R | insulin like growth factor 1 receptor | Plasma Membrane | transmembrane receptor |
| 51 | IGF2BP1 | insulin like growth factor 2 mRNA binding protein 1 | Cytoplasm | translation regulator |
| 52 | IGF2BP2 | insulin like growth factor 2 mRNA binding protein 2 | Cytoplasm | translation regulator |
| 53 | IGF2BP3 | insulin like growth factor 2 mRNA binding protein 3 | Cytoplasm | translation regulator |
| 54 | IGFBP5 | insulin like growth factor binding protein 5 | Extracellular Space | other |
| 55 | IKZF1 | IKAROS family zinc finger 1 | Nucleus | transcription regulator |
| 56 | IL6 | interleukin 6 | Extracellular Space | cytokine |
| 57 | ITGA5 | integrin subunit alpha 5 | Plasma Membrane | transmembrane receptor |
| 58 | ITGB3 | integrin subunit beta 3 | Plasma Membrane | transmembrane receptor |
| 59 | KIF23 | kinesin family member 23 | Cytoplasm | other |
| 60 | KITLG | KIT ligand | Extracellular Space | growth factor |
| 61 | KRAS | KRAS proto-oncogene, GTPase | Cytoplasm | enzyme |
| 62 | MAP4K4 | mitogen-activated protein kinase kinase kinase kinase 4 | Cytoplasm | kinase |
| 63 | MAPK3 | mitogen-activated protein kinase 3 | Cytoplasm | kinase |
| 64 | MAPK7 | mitogen-activated protein kinase 7 | Cytoplasm | kinase |
| 65 | MAPRE1 | microtubule associated protein RP/EB family member 1 | Cytoplasm | other |
| 66 | MDM2 | MDM2 proto-oncogene | Nucleus | transcription regulator |
| 67 | MEF2C | myocyte enhancer factor 2C | Nucleus | transcription regulator |
| 68 | MICA | MHC class I polypeptide-related sequence A | Plasma Membrane | other |
| 69 | MMP3 | matrix metallopeptidase 3 | Extracellular Space | peptidase |
| 70 | MYC | v-myc avian myelocytomatosis viral oncogene homolog | Nucleus | transcription regulator |
| 71 | NF1 | neurofibromin 1 | Cytoplasm | other |
| 72 | NFIA | nuclear factor I A | Nucleus | transcription regulator |
| 73 | OSBPL2 | oxysterol binding protein like 2 | Cytoplasm | other |
| 74 | OSBPL8 | oxysterol binding protein like 8 | Plasma Membrane | transporter |
| 75 | PCGF1 | polycomb group ring finger 1 | Nucleus | other |
| 76 | PDCD4 | programmed cell death 4 (neoplastic transformation inhibitor) | Nucleus | other |
| 77 | PDPK1 | 3-phosphoinositide dependent protein kinase 1 | Cytoplasm | kinase |
| 78 | PIK3R1 | phosphoinositide-3-kinase regulatory subunit 1 | Cytoplasm | kinase |
| 79 | PKD2 | polycystin 2, transient receptor potential cation channel | Plasma Membrane | ion channel |
| 80 | PLK1 | polo like kinase 1 | Nucleus | kinase |
| 81 | PMAIP1 | phorbol-12-myristate-13-acetate-induced protein 1 | Cytoplasm | other |
| 82 | PPARG | peroxisome proliferator activated receptor gamma | Nucleus | ligand-dependent nuclear receptor |
| 83 | PRC1 | protein regulator of cytokinesis 1 | Nucleus | other |
| 84 | PRDM1 | PR domain 1 | Nucleus | transcription regulator |
| 85 | PTEN | phosphatase and tensin homolog | Cytoplasm | phosphatase |
| 86 | PXN | paxillin | Cytoplasm | other |
| 87 | RAF1 | Raf-1 proto-oncogene, serine/threonine kinase | Cytoplasm | kinase |
| 88 | RB1 | RB transcriptional corepressor 1 | Nucleus | transcription regulator |
| 89 | RECK | reversion inducing cysteine rich protein with kazal motifs | Plasma Membrane | other |
| 90 | RFFL | ring finger and FYVE-like domain containing E3 ubiquitin protein ligase | Cytoplasm | enzyme |
| 91 | RUNX1 | runt related transcription factor 1 | Nucleus | transcription regulator |
| 92 | RUNX2 | runt related transcription factor 2 | Nucleus | transcription regulator |
| 93 | SERPINB5 | serpin family B member 5 | Extracellular Space | other |
| 94 | SLC12A1 | solute carrier family 12 member 1 | Plasma Membrane | transporter |
| 95 | SOD3 | superoxide dismutase 3, extracellular | Extracellular Space | enzyme |
| 96 | SPI1 | Spi-1 proto-oncogene | Nucleus | transcription regulator |
| 97 | SPRY1 | sprouty RTK signaling antagonist 1 | Cytoplasm | other |
| 98 | Spry1 | sprouty RTK signaling antagonist 1 | Cytoplasm | other |
| 99 | SRF | serum response factor | Nucleus | transcription regulator |
| 100 | STAT3 | signal transducer and activator of transcription 3 | Nucleus | transcription regulator |
| 101 | TCF21 | transcription factor 21 | Nucleus | transcription regulator |
| 102 | TGFBR1 | transforming growth factor beta receptor 1 | Plasma Membrane | kinase |
| 103 | TGFBR2 | transforming growth factor beta receptor 2 | Plasma Membrane | kinase |
| 104 | THRB | thyroid hormone receptor beta | Nucleus | ligand-dependent nuclear receptor |
| 105 | TIMP3 | TIMP metallopeptidase inhibitor 3 | Extracellular Space | other |
| 106 | TLR3 | toll like receptor 3 | Plasma Membrane | transmembrane receptor |
| 107 | TNF | tumor necrosis factor | Extracellular Space | cytokine |
| 108 | TOP2A | topoisomerase (DNA) II alpha | Nucleus | enzyme |
| 109 | TP63 | tumor protein p63 | Nucleus | transcription regulator |
| 110 | TPM1 | tropomyosin 1 (alpha) | Cytoplasm | other |
| 111 | TRPS1 | transcriptional repressor GATA binding 1 | Nucleus | transcription regulator |
| 112 | TWF1 | twinfilin actin binding protein 1 | Cytoplasm | kinase |
| 113 | UCP2 | uncoupling protein 2 | Cytoplasm | transporter |
| 114 | WEE1 | WEE1 G2 checkpoint kinase | Nucleus | kinase |
| 115 | WNT3A | Wnt family member 3A | Extracellular Space | cytokine |
| 116 | ZEB1 | zinc finger E-box binding homeobox 1 | Nucleus | transcription regulator |
| 117 | ZEB2 | zinc finger E-box binding homeobox 2 | Nucleus | transcription regulator |
| 118 | ZYX | zyxin | Plasma Membrane | other |
