# Supplementary material for: Characterization of mouse serum exosomal small RNA content: The origins and their roles in modulating inflammatory response
Source: Oncotarget. 2017 Apr 27;8(26):42712–27. doi: 10.18632/oncotarget.17448 (PMC5522100; doi:10.18632/oncotarget.17448)
Supplement: Supplementary file 4 [file oncotarget-08-42712-s004.docx]

|  | **Categories** | **p-Value** | **Molecules** | **# Molecules** |
| --- | --- | --- | --- | --- |
| 1 | Cancer, Hematological Disease, Immunological Disease, Organismal Injury and Abnormalities | 1.13E-24 | let-7a-5p (and other miRNAs w/seed GAGGUAG),miR-10a-5p (and other miRNAs w/seed ACCCUGU),miR-140-3p (and other miRNAs w/seed ACCACAG),miR-148a-3p (and other miRNAs w/seed CAGUGCA),miR-16-5p (and other miRNAs w/seed AGCAGCA),miR-181a-5p (and other miRNAs w/seed ACAUUCA),miR-192-5p (and other miRNAs w/seed UGACCUA),miR-21-5p (and other miRNAs w/seed AGCUUAU),miR-22-3p (miRNAs w/seed AGCUGCC),miR-26a-5p (and other miRNAs w/seed UCAAGUA),miR-30c-5p (and other miRNAs w/seed GUAAACA),miR-92a-3p (and other miRNAs w/seed AUUGCAC) | 12 |
| 2 | Cancer, Organismal Injury and Abnormalities, Reproductive System Disease | 1.55E-16 | let-7a-5p (and other miRNAs w/seed GAGGUAG),miR-10a-5p (and other miRNAs w/seed ACCCUGU),miR-16-5p (and other miRNAs w/seed AGCAGCA),miR-181a-5p (and other miRNAs w/seed ACAUUCA),miR-192-5p (and other miRNAs w/seed UGACCUA),miR-21-5p (and other miRNAs w/seed AGCUUAU),miR-26a-5p (and other miRNAs w/seed UCAAGUA),miR-30c-5p (and other miRNAs w/seed GUAAACA),miR-92a-3p (and other miRNAs w/seed AUUGCAC) | 9 |
| 3 | Cancer, Gastrointestinal Disease, Organismal Injury and Abnormalities, Respiratory Disease | 2.44E-16 | let-7a-5p (and other miRNAs w/seed GAGGUAG),miR-1-3p (and other miRNAs w/seed GGAAUGU),miR-10a-5p (and other miRNAs w/seed ACCCUGU),miR-126a-5p (and other miRNAs w/seed AUUAUUA),miR-143-3p (and other miRNAs w/seed GAGAUGA),miR-16-5p (and other miRNAs w/seed AGCAGCA),miR-21-5p (and other miRNAs w/seed AGCUUAU),miR-26a-5p (and other miRNAs w/seed UCAAGUA),miR-27a-3p (and other miRNAs w/seed UCACAGU),miR-30c-5p (and other miRNAs w/seed GUAAACA) | 10 |
| 4 | Cancer, Gastrointestinal Disease, Organismal Injury and Abnormalities, Respiratory Disease | 3.28E-16 | let-7a-5p (and other miRNAs w/seed GAGGUAG),miR-1-3p (and other miRNAs w/seed GGAAUGU),miR-10a-5p (and other miRNAs w/seed ACCCUGU),miR-126a-5p (and other miRNAs w/seed AUUAUUA),miR-143-3p (and other miRNAs w/seed GAGAUGA),miR-16-5p (and other miRNAs w/seed AGCAGCA),miR-21-5p (and other miRNAs w/seed AGCUUAU),miR-26a-5p (and other miRNAs w/seed UCAAGUA),miR-30c-5p (and other miRNAs w/seed GUAAACA) | 9 |
| 5 | Cancer, Connective Tissue Disorders, Organismal Injury and Abnormalities | 3.34E-15 | let-7a-5p (and other miRNAs w/seed GAGGUAG),miR-143-3p (and other miRNAs w/seed GAGAUGA),miR-146a-5p (and other miRNAs w/seed GAGAACU),miR-16-5p (and other miRNAs w/seed AGCAGCA),miR-21-5p (and other miRNAs w/seed AGCUUAU),miR-26a-5p (and other miRNAs w/seed UCAAGUA),miR-451a (and other miRNAs w/seed AACCGUU),miR-486-5p (and other miRNAs w/seed CCUGUAC) | 8 |
| 6 | Cancer, Hematological Disease, Immunological Disease, Organismal Injury and Abnormalities | 6.61E-15 | let-7a-5p (and other miRNAs w/seed GAGGUAG),miR-10a-5p (and other miRNAs w/seed ACCCUGU),miR-140-3p (and other miRNAs w/seed ACCACAG),miR-143-3p (and other miRNAs w/seed GAGAUGA),miR-148a-3p (and other miRNAs w/seed CAGUGCA),miR-16-5p (and other miRNAs w/seed AGCAGCA),miR-181a-5p (and other miRNAs w/seed ACAUUCA),miR-192-5p (and other miRNAs w/seed UGACCUA),miR-21-5p (and other miRNAs w/seed AGCUUAU),miR-22-3p (miRNAs w/seed AGCUGCC),miR-26a-5p (and other miRNAs w/seed UCAAGUA),miR-30c-5p (and other miRNAs w/seed GUAAACA),miR-451a (and other miRNAs w/seed AACCGUU),miR-92a-3p (and other miRNAs w/seed AUUGCAC) | 14 |
| 7 | Cancer, Connective Tissue Disorders, Organismal Injury and Abnormalities | 2.9E-13 | miR-143-3p (and other miRNAs w/seed GAGAUGA),miR-146a-5p (and other miRNAs w/seed GAGAACU),miR-16-5p (and other miRNAs w/seed AGCAGCA),miR-21-5p (and other miRNAs w/seed AGCUUAU),miR-26a-5p (and other miRNAs w/seed UCAAGUA),miR-451a (and other miRNAs w/seed AACCGUU),miR-486-5p (and other miRNAs w/seed CCUGUAC) | 7 |
| 8 | Inflammatory Response | 2.89E-11 | let-7a-5p (and other miRNAs w/seed GAGGUAG),miR-126a-5p (and other miRNAs w/seed AUUAUUA),miR-140-3p (and other miRNAs w/seed ACCACAG),miR-143-3p (and other miRNAs w/seed GAGAUGA),miR-151-3p (and other miRNAs w/seed UAGACUG),miR-16-5p (and other miRNAs w/seed AGCAGCA),miR-181a-5p (and other miRNAs w/seed ACAUUCA),miR-21-5p (and other miRNAs w/seed AGCUUAU),miR-22-3p (miRNAs w/seed AGCUGCC),miR-26a-5p (and other miRNAs w/seed UCAAGUA),miR-27a-3p (and other miRNAs w/seed UCACAGU),miR-30c-5p (and other miRNAs w/seed GUAAACA),miR-486-5p (and other miRNAs w/seed CCUGUAC),miR-92a-3p (and other miRNAs w/seed AUUGCAC) | 14 |
| 9 | Cancer, Organismal Injury and Abnormalities | 4.1E-11 | let-7a-5p (and other miRNAs w/seed GAGGUAG),miR-10a-5p (and other miRNAs w/seed ACCCUGU),miR-146a-5p (and other miRNAs w/seed GAGAACU),miR-148a-3p (and other miRNAs w/seed CAGUGCA),miR-27a-3p (and other miRNAs w/seed UCACAGU),miR-92a-3p (and other miRNAs w/seed AUUGCAC) | 6 |
